# Influence of cultivar, fruit maturity, and harvest month on the targeted metabolite profile of Highland strawberries (*Fragaria* × *ananassa* Duch.)

**DOI:** 10.1016/j.fochx.2026.104077

**Published:** 2026-06-06

**Authors:** Ji-Ye Kim, Muthu Thiruvengadam, Doyeon Kim, Hee-Youn Chi, Hee-Jin Choi, Ja-Min Lee, Dagyeom Jeon, Yunwoo Park, Seung-Hyun Kim

**Affiliations:** a Department of Crop Science, College of Life Science, Konkuk University, Seoul 05029, Republic of Korea; b National Institute of Crop Science, Rural Development Administration, Jeonju-Si 54875, Republic of Korea

**Keywords:** Strawberry, Day-neutral and everbearing, Vitamin C, B-complex vitamins, Targeted phenolics, UPLC–MS/MS

## Abstract

The development of everbearing (Jangha and Miha) and day-neutral (Goseul) strawberry cultivars has enabled extended highland production; however, seasonal and maturity-driven metabolite variations remain unclear. This study quantified vitamin C, B-complex vitamins, and phenolics from August to October at 60% and 100% ripeness. The total vitamin C content ranged from 4.4 to 6.3 mg·g^−1^ dw in Goseul, 5.5 to 7.2 mg·g^−1^ dw in Jangha, and 5.5 to 8.8 mg·g^−1^ dw in Miha, with significant increases across harvest months in Goseul and Jangha (*p* < 0.05). Targeted phenolic compounds increased significantly at full ripeness (*p* < 0.05), with the highest 6.3-fold increase observed in Goseul harvested in October (3132.43 vs. 497.87 μg·g^−1^ dw), mainly due to pelargonidin 3-O-glucoside. Total B-complex vitamin levels decreased significantly by up to 53% across three cultivars as they ripened. Overall, fruit maturity had a stronger influence on metabolite composition than harvest timing.

## Highlights

• Metabolite profiles of highland strawberry varied with cultivar and fruit maturity.
• Fruit maturity dominated phenolic and B-vitamin variation more than harvest month.
• Phenolics increased 2–3× at full ripeness, driven by pelargonidin 3-O-glucoside.
• B vitamin content was higher in immature fruits.
• Day-neutral and ever-bearing cultivars showed distinct seasonal phenolic responses.

## 1 Introduction

Strawberry (*Fragaria* × *ananassa* Duch.) is among the most popular fruits worldwide, with a global production of approximately 12.2 million tons in 2020, according to the Food and Agriculture Organization (FAO (Food and Agriculture Organization of the United Nations), 2023). The global fresh strawberry market, valued at USD 19 billion in 2024, is projected to expand to USD 31.82 billion by 2034, indicating a stronger trend (EMR (Expert Market Research), 2024; Eum et al., 2025). Strawberry is an herbaceous, perennial, and stoloniferous species of the Rosaceae family, with over 20 wild *Fragaria* species distributed worldwide. Its fruit has high economic value in the global market because of its organoleptic properties and strong nutritional profile (Fernández-Veloso et al., 2025). Cultivated strawberries are primarily octoploid (8×) and constitute the most widely produced berry crop worldwide, with China accounting for approximately 40% of the global production, according to FAO statistics (FAO (Food and Agriculture Organization of the United Nations), 2023; Hernández-Martínez et al., 2023). Strawberries are among the highest-valued commodities in South Korea, accounting for nearly 1.5 trillion KRW in 2023 (FAO (Food and Agriculture Organization of the United Nations), 2023). In addition to being commonly consumed fresh, strawberries are widely used in processed foods such as ice cream, jams, juices, jellies, pastry cakes, and cosmetic products (Charoenwoodhipong et al., 2025). In addition to their desirable flavor and aroma, strawberries are rich in bioactive compounds, particularly phenolic compounds, anthocyanins, and vitamin C, which contribute to their strong antioxidant potential (Duan, Bao, Jiang, et al., 2025, Duan, Wang, Song, et al., 2025). Strawberries contain approximately 58.8 mg of vitamin C per 100 g fresh weight (USDA (United States Department of Agriculture), 2019). Their antioxidant activity is further enhanced by the synergistic interactions between vitamin C and polyphenols (Giampieri et al., 2012). Anthocyanins, particularly pelargonidin-based derivatives, are the major polyphenols responsible for the characteristic red color of strawberries fruits (Martínez-Ferri et al., 2023). Owing to their rich phytochemical composition, strawberries are increasingly regarded as functional fruits with high nutritional and commercial values.

Strawberry quality and functional metabolite profiles are influenced by cultivar, ripening stage, harvest time, and storage conditions (Kim et al., 2023; Milosavljević et al., 2023; Pedrozo et al., 2024). For example, anthocyanin levels generally peak at full maturity, increasing several-fold relative to those in immature fruits; however, fruit firmness and susceptibility to post-harvest damage vary with fruit ripeness (Skrovankova et al., 2015; Wang et al., 2025). To extend shelf life, strawberries are often harvested at partial ripeness, typically 50–70% in spring and 80–90% in winter (Taghavi et al., 2019; Hong & Eum, 2020). Strawberries are commonly categorized into three photoperiod-based classifications: June-bearing (short-day), everbearing, and day-neutral types. June-bearing cultivars initiate flowering under short-day conditions, whereas everbearing types produce two to three flushes of fruit under specific photoperiod and temperature conditions (Park et al., 2023). In contrast, day-neutral cultivars are capable of continuous flowering and fruiting, irrespective of day length, although temperature remains a limiting factor (Darby & Islam, 2025). In this study, day-neutral and everbearing types were treated as distinct categories based on their physiological responses to the photoperiod and fruiting behavior, despite occasional grouping in some classification systems. The selected cultivars, ‘Jangha’ and ‘Miha’ (everbearing types) and ‘Goseul’ (day-neutral type), were chosen to represent the distinct flowering habits and genetic backgrounds commonly utilized in summer highland strawberry production systems in South Korea. Everbearing cultivars, such as ‘Jangha’ and ‘Miha’ exhibit repeated flowering and fruiting cycles under favorable environmental conditions, whereas the day-neutral cultivar ‘Goseul’ can flower continuously regardless of day length. These physiological and genetic differences are expected to influence assimilate partitioning, fruit development, and the accumulation of bioactive metabolites during ripening. Compared with short-day cultivars, everbearing and day-neutral cultivars generally produce smaller fruits owing to continuous flowering and fruit set, making them suitable for processing and specialty market applications (Lee et al., 2017; Lee et al., 2020; Lee et al., 2024). In contrast, short-day cultivars dominate commercial production owing to their higher yields, larger fruit sizes, and superior market preferences (Rutz et al., 2023). Among these, Albion (day-neutral), Camarosa, and Festival (short-day) are widely cultivated strawberry cultivars, particularly in major producing countries such as the United States, Spain, and China (FAO (Food and Agriculture Organization of the United Nations), 2023). For example, Camarosa has historically been favored for its high productivity and adaptability (Chiomento et al., 2021), whereas Albion is increasingly preferred for its fruit quality and extended shelf-life (Park et al., 2023). Collectively, these differences demonstrate the importance of cultivar-specific physiological traits in determining fruit quality and its market value. Therefore, the inclusion of contrasting everbearing and day-neutral cultivars in the present study provides a valuable framework for evaluating variations in nutritional metabolites and antioxidant potential during fruit ripening in strawberries.

Despite the increasing global cultivation of everbearing and day-neutral strawberry cultivars, existing research has mainly focused on agronomic performance, including growth characteristics, yield optimization, and adaptation to highland or controlled environment cultivation systems. Durner et al. (1984) and Samad et al. (2021) investigated the effects of temperature and photoperiod on flowering behavior and yield in day-neutral and everbearing strawberry cultivars, respectively. Cervantes et al. (2020) assessed the morphological characteristics, fruit quality, fruit production, and yield stability of strawberry cultivars. Although these studies have significantly contributed to enhancing productivity, comparatively little attention has been paid to the systematic characterization of the functional and nutritional metabolite profiles of these cultivars. In particular, there is a lack of comprehensive studies addressing (i) changes in essential bioactive compounds, including anthocyanins, flavonoids, phenolic acids, vitamin C, and B-complex vitamins, among cultivars; (ii) temporal changes in metabolite accumulation across consecutive harvest months under varying environmental conditions; and (iii) dynamic metabolic alterations associated with fruit maturity stages in short-day, day-neutral, and everbearing strawberries. Moreover, most previous studies have focused on single harvest points or cultivars at limited developmental stages cultivars (Chen et al., 2024; Kim et al., 2023; Wang et al., 2023), thereby overlooking the combined effects of genotype, harvest timing, and ripening stage on metabolite composition and antioxidant potential. Based on these gaps, the present study was designed to address the following research question: how do cultivar type (everbearing vs. day-neutral), harvest month, and fruit maturity interact to influence the accumulation of vitamin C, B-complex vitamins, and phenolic compounds in strawberries grown under highland summer conditions? This study hypothesized that fruit maturity is the primary determinant of phenolic accumulation, with higher concentrations at full ripeness. B-complex vitamins would decrease with increasing ripeness due to metabolic utilization during fruit development, and cultivar-specific responses would modulate the magnitude and pattern of metabolite changes across the harvest months. This study aimed to assess the vitamin C, B-complex vitamin, and targeted phenolic profiles of three summer-grown strawberry cultivars (Jangha, Miha and Goseul) across harvest months (August–October) and at two maturity stages (60% and 100%). These findings provide a mechanistic basis for optimizing cultivar selection, harvest timing, and cultivation strategies to enhance the nutritional quality of strawberries.

## 2 Materials and methods

### 2.1 Sample preparation

**Fig. 1:**
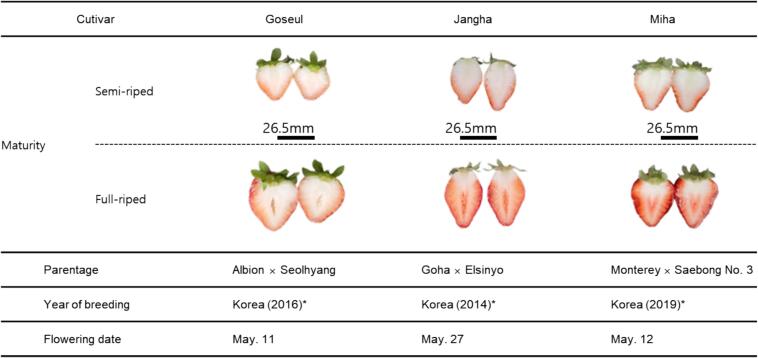
Information on the ever-bearing and day-neutral strawberry cultivars examined in this study. * = Rural development administration.

Fruit samples were collected from two everbearing strawberry cultivars (Jangha and Miha) and a day-neutral cultivar (Goseul). The plants were cultivated in 2023 at the Rural Development Administration, National Institute of Highland Agriculture, Pyeongchang, Korea (37°40′52″N, 128°43′53″E; elevation 785 m above sea level). During the cultivation period, the experimental site experienced typical highland climatic conditions, with a monthly mean temperature ranging from (13.2–23.8 °C), and relative humidity maintained at 54.7–80.8% under greenhouse conditions (Table S1). Fig. 1 presents the three strawberry cultivars examined in this study. Overwintered seedlings established in the field were transferred in April 2023 to a rain shelter house (8 × 60 m; elevation 785 m) and transplanted into Cocogold slabs (90 × 20 × 10 cm; 100% dust; Daeyoung GS, Korea) using a two-plot system. The experiment followed a completely randomized block design, with three replicates, each comprising 10 plants. Plants were fertigated with Dutch PBG nutrient solution (N–P–K–Ca–Mg–S = 12.5–3.0–5.5–6.5–2.5–3.0 me∙L^−1^). Nutrient concentrations and application volumes were adjusted according to the growth stage. Electrical conductivity was maintained at 0.6–1.5 dS∙m^−1^, and 60 mL of solution was supplied 3–7 times daily. The pH ranged was maintained at 5.5–6.5.

Strawberries were harvested on the 2nd, 12th, and 24th of each month from August to October. To assess maturity effects, fruits were classified as immature (approximately 60% surface red pigmentation) or mature (100% full red pigmentation) based on standardized visual assessment criteria to ensure consistency across samples. To minimize the effect of short-term variability and obtain a representative monthly profile, data from the three harvest dates were pooled and analyzed as a single composite monthly dataset. At each sampling date, approximately 30 fruits per cultivar were collected. From these, ~10 fruits of uniform size and shape were selected, de-stemmed, cut into small pieces, and immediately frozen at −80 °C (U700, Thermo Fisher Scientific, USA). Subsequently, all fruits collected within the same month (~30 fruits per cultivar) were pooled and prepared as three biological replicates per cultivar per maturity per month and these pooled samples were stored at −80 °C for approximately 5 months up to metabolite analysis to preserve biochemical integrity. Prior to chemical analysis, each pooled sample (7 g) was then lyophilized at −85 °C for 72 h (FM25EL, Virtis, USA), ground using a mortar and pestle, and stored in a desiccator (room temperature, 11–15% RH). To ensure stability and minimize degradation, vitamin C and B-complex compounds were analyzed within 3 days of processing, while phenolic compounds were analyzed within one week of pulverization.

### 2.2 Chemicals and reagents

All solvents and reagents used in this study were of analytical or high-performance liquid chromatography (HPLC) grade. Acetonitrile (ACN) and methanol (MeOH) were purchased from Thermo Fisher Scientific (Seoul, Korea). Deionized water (DIW) was produced using a PURELAB Optio system (ELGA Lab Water, UK) and used throughout the experiments (resistivity >17 MΩ). Formic acid was acquired from Junsei (Tokyo, Japan), 0.1 N hydrochloric acid (HCl) from Daejung Chemicals & Metals Co. Ltd. (Gyeonggi-Do, Korea), sulfuric acid (H_2_SO_4_) from Baker (Pennsylvania, USA), and sodium hydroxide (NaOH) from Samchun Chemical Co., Ltd. (Gyeonggi-do, Korea). Ammonium acetate, metaphosphoric acid (HPO_3_), dimethyl sulfoxide (DMSO), 2-amino-2-hydroxymethyl-1,3-propanediol (Tris), and dithiothreitol (DTT) were purchased from Sigma-Aldrich (St. Louis, MO, USA). Ascorbic acid, vitamin B complex, and 59 phenolic standards were acquired from Sigma-Aldrich Corp. (St. Louis, MO, USA), Wako Pure Chemical Industries (Osaka, Japan), Cayman Chemical (Ann Arbor, MI, USA), and LC Laboratories (Woburn, MA, USA).

### 2.3 Vitamin C analysis

Vitamin C was extracted using an ultrasound-assisted extraction (UAE) method (Kim et al., 2025). Total vitamin C was quantified as the sum of ascorbic acid (AA) and dehydroascorbic acid (DHAA). For extraction, 100 mg of freeze-dried strawberry powder was mixed with 10 mL of 1% HPO_3_ and vortexed briefly (5 s). The mixture was sonicated for 10 min at 340 W (<45 °C) using an ultrasonic processor (JAC-5020; 40 kHz; U1tech, Gyeonggi-do, Korea). The sample tubes were repositioned every 3 min to ensure uniform extraction. After centrifugation (2200 ×*g*, 10 min, 4 °C), the supernatant was filtered through a 0.20 μm hydrophilic PTFE syringe filter (ADVANTEC) into amber vials for AA analysis. Because DHAA cannot be directly detected by HPLC-DAD, it was reduced to AA prior to analysis. For total ascorbic acid (TAA) determination, 90 μL of the extract was mixed with 90 μL of 40 mM DTT prepared in 0.5 M Tris buffer (pH 9.0). After gentle mixing (5 s), the reaction proceeded in the dark for 30 min and was then quenched with 90 μL of 0.4 M H₂SO_4_. The DHAA content was calculated as the difference between the TAA and AA contents.

TAA (mg∙g^−1^) = DHAA (mg∙g^−1^) + AA (mg∙g^−1^).

Vitamin C (AA + dehydroascorbic acid) was quantified using an ultrahigh-performance liquid chromatography system (UHPLC 1290 Infinity, Agilent Technologies, Santa Clara, CA, USA) equipped with a diode array detector (DAD). Separation was performed on a Shim-pack GIST C18 column (2.1 × 100 mm, 2 μm) coupled to an Eclipse Plus C18 guard column (2.1 × 5 mm, 1.8 μm). The mobile phases consisted of solvent A (0.1% formic acid in DIW) and solvent B (0.1% formic acid in ACN). The gradient program was as follows: 0 min, 95% A; 1 min, 85% A; 2 min, 65% A; 6 min, 95% A. The flow rate was 0.3 mL·min^−1^, the injection volume was 5 μL, and the column temperature was ambient. The autosampler was maintained at 4 °C to minimize analyte oxidation. Vitamin C was monitored at 245 nm, and spectral data (190–640 nm, 2-nm intervals) were collected to confirm peak identity. The total run time was 7 min per sample (6 min analysis and 1 min post-run). To prevent vitamin C oxidation, a new solution (100 μg·mL^−1^) was prepared in 1% HPO_3_. Calibration was performed using serial dilutions (5, 10, 15, 20, 25, 30, and 40 μg·mL^−1^). Each concentration was injected in duplicate, and mean peak areas were used to construct the calibration curve, which exhibited excellent linearity (R^2^ = 0.9997) and the calibration curve accuracy of 101 ± 2%, calculated as the ratio of the measured concentration to the nominal (theoretical) concentration across the calibration range (5–40 μg·mL^−1^). Further, a limit of detection (LOD) and quantification (LOQ) based on this calibration curve were 1.43 ppm (μg·mL^−1^) and 4.32 ppm (μg·mL^−1^), respectively (Table S2).

### 2.4 Vitamin B-complex analysis

The B vitamins (B1, B2, B3-acid, B3-amide, B5, B6, B7, B9, and B12) were extracted using UAE (Kim et al., 2025). Briefly, 100 mg of dried strawberry powder was mixed with 10 mL of an extraction solvent comprising 2.5 mL of DIW and 7.5 mL of 5 mM ammonium acetate containing 0.1% (*v*/v) of formic acid. After stirring for 5 s, the mixture was ultrasonically extracted for 30 min at 340 W (< 40 °C) using an ultrasonic cleaner (JAC-5020, 40 kHz; U1tech, Gyeonggi-do, Korea). The sample positions within the ultrasonic bath were randomized every 3 min to ensure uniform extraction. The resulting mixture was centrifuged at 2200 ×*g* for 10 min at 4 °C, and the supernatant was filtered through a 0.20 μm hydrophilic PTFE syringe filter (13 mm, ADVANTEC) into 1.5 mL amber vials for vitamin B-complex analysis.

The vitamin B-complex compounds were quantified using a UHPLC system (Nexera X3, Shimadzu, Japan) coupled to a triple quadrupole mass spectrometer (LC-MS 8050, Shimadzu, Japan). The analytes were ionized in the positive electrospray ionization mode (+ESI) and detected by multiple reaction monitoring (MRM). Chromatographic separation was performed on a Shim-pack GIST C18 column (2.1 × 100 mm, 2 μm; Shimadzu) maintained at 40 °C. The mobile phases consisted of solvent A (5 mM ammonium acetate in 0.1% formic acid in DIW) and solvent B (100% MeOH), and gradient elution was set as follows: 0 min, 100% A; 2 min, 100% A; 2.5 min, 70% A; 5 min, 50% A; 5.5 min, 1% A; 7 min, 1% A; 7.1 min, 100% A; 12 min, 100% A. The flow rate was 0.3 mL·min^−1^, the injection volume was 5 μL, and the autosampler temperature was 15 °C. Nitrogen generated by a nitrogen generator (AT-ADVANCE 10–5, Shimadzu) was used as the nebulizing, heating, and drying gas (neb., 3 L·min^−1^; heating and drying, 10 L·min^−1^). Argon was used as the collision gas in Q2. The interface, desolvation line, and heat block temperatures were 300, 250, and 400 °C, respectively. The total run time was 12 min per sample. The MRM transitions and optimized MS parameters for each vitamin are summarized in Table S3. Standard solutions (100 μg·mL^−1^) were freshly prepared prior to analysis. Vitamins B1, B3-acid, B3-amide, B5, B6, and B12 were dissolved and diluted in solvent A, whereas vitamins B2, B7, and B9 were initially dissolved in 0.01 M NaOH and subsequently diluted in solvent A. Calibration curves were generated by stepwise dilution, with each level analyzed in duplicate. Quantification was based on the mean peak area. The accuracy of calibration curves for the 7 vitamin B complex ranged from 91% to 104% with confirmed linearity (R^2^ > 0.97); and the LOD/LOQ and repeatability were summarized in Table S4.

### 2.5 Targeted phenolic compounds analysis

Phenolic compounds were extracted as described by Kim et al. (2023) with minor modifications. Briefly, 50 mg of freeze-dried strawberry powder was mixed with 10 mL of ACN and 2 mL of 0.1 N HCl in a conical flask and shaken at 200 rpm for 2 h at room temperature (Green-SSeriker; Vision Scientific Co. Ltd., Gyeonggi-do, Korea). The mixture was filtered through a Whatman No. 42 filter paper (110 mm diameter) into a 250 mL round-bottom flask, and the filtrate was concentrated under reduced pressure at <35 °C using a rotary evaporator (EYELA SB-1200, Tokyo Rikakikai Co., Tokyo, Japan). The dried residue was reconstituted in 5 mL of 80% MeOH, filtered through a 0.20 μm PTFE syringe filter (13 mm, hydrophobic; Whatman), and transferred to a 1.5 mL vial for further analysis.

Targeted phenolic metabolites (59 compounds) were quantified using UHPLC–MS/MS under the general instrumental conditions described in Section 2.4. The samples were analyzed in both positive and negative electrospray ionization (ESI) modes using the MRM technique. Chromatographic separation was performed on a reverse-phase C18 column (Shim-pack GIST, 2.1 × 100 mm, 2 μm; Shimadzu) maintained at 40 °C. The mobile phase consisted of solvent A (0.1% formic acid in DIW) and solvent B (0.1% formic acid in ACN), and gradient elution was set as follows: 0 min, 90% A; 10 min, 30% A; 12 min, 0% A; 13 min, 0% A; 15 min, 90% A; 20 min, 90% A. The flow rate was 0.3 mL·min^−1^ and the injection volume was 5 μL. The gas and auto-sampler conditions were identical to those used for the vitamin B-complex analysis. The total runtime per sample was 20 min. The compound-specific MRM transitions and MS parameters are listed in Table S5. Stock solutions of each phenolic standard (1000 μg·mL^−1^) were prepared in 80% MeOH. When required, standards were dissolved in DMSO and then diluted with 80% MeOH. Calibration standards were prepared by serial dilution, analyzed in duplicate and averaged. The calibration curves for the 19 phenolics showed good accuracy (95–108%) and excellent linearity (R^2^ > 0.99); the LOD/LOQ and repeatability of these calibration curves were also shown in Table S6.

### 2.6 Statistical analysis

All experiments were performed in triplicate (*n* = 3), and the results are presented as mean ± standard deviation (SD). Statistical analyses were performed using SAS software (version 9.4; SAS Institute, Inc., Cary, NC, USA). Correlations were assessed at a significance level of *p* < 0.05. Three-way ANOVA was performed using IBM SPSS Statistics (version 25; IBM Corp., Armonk, NY, USA), followed by Tukey's and the least significant difference (LSD) tests for mean separation (*p* < 0.05). Principal component analysis (PCA) was performed using the MetaboAnalyst 6.0 software. Specifically, the sample group was normalized using median imputation, and the dataset was subsequently subjected to autoscaling (mean centering followed by division by the standard deviation of each variable).

## 3 Results and discussion

### 3.1 Influence of cultivars, fruit maturity, and harvest month on variations in vitamin C

Vitamin C levels varied significantly across cultivars and harvest months, highlighting the strong genotype-dependent metabolism and seasonal environmental effects on antioxidant accumulation (Lee & Kader, 2000). As shown in Table 1, a three-way analysis of variance (ANOVA) revealed that both cultivar (C) and harvest month (H) exerted highly significant effects on ascorbic acid (AA), dehydroascorbic acid (DHAA), and total ascorbic acid (TAA) (*p* < 0.001), with partial η^2^ values ranging from 0.456 to 0.784. According to Cohen's effect size criteria for partial η^2^ (0.01 = small, 0.06 = medium, and 0.14 = large), all observed effects can be classified as large, indicating substantial contributions of these factors to the variation in the vitamin C components. Notably, the harvest month demonstrated the strongest influence on AA (η^2^ = 0.784), while the cultivar showed a similarly strong contribution to TAA variation (η^2^ = 0.768), indicating that both seasonal and genetic factors play dominant roles in determining the vitamin C content in strawberries. Although fruit maturity (M) did not exert a significant overall main effect on vitamin C concentrations (*p* > 0.05), with negligible effect sizes (η^2^ = 0.005–0.096), the response to maturity varied among cultivars, indicating a potential cultivar × maturity interaction. At the cultivar-specific (simple effect) level, the ‘Miha’ cultivar exhibited increased vitamin C levels at the semi-ripe stage, whereas this trend was not consistently observed in the other cultivars (Table S2). These findings suggest that maturity-related changes in vitamin C accumulation are genotype-dependent and may be masked when considering the overall effect of maturity alone. These strong main effects indicate substantial genotypic variation and seasonal influences on ascorbate metabolism, consistent with previous findings for strawberries (Van de Velde et al., 2012). Vitamin C content was significantly influenced by cultivar type and harvest timing. Early season harvests of short-day (Aguedilla and Sabrosa) cultivars exhibited the highest vitamin C concentrations, while late-harvested short-day (Camarosa and Fuentepina) cultivars showed the lowest levels (Domínguez et al., 2016). Significant C × H interactions were observed for all vitamin C components (*p* < 0.05 to *p* < 0.001; η^2^ = 0.232–0.622), indicating that the influence of cultivar varied depending on harvest timing. Additionally, the three-way interaction (C × H × M) was significant for AA, DHAA, and TAA (η^2^ = 0.239–0.406), further supporting a combined modulatory effect of genotype and seasonal factors, although maturity alone remained a weak contributor. These findings collectively demonstrate that cultivar selection and harvest timing are the primary determinants of vitamin C variability, whereas fruit maturity plays a comparatively minor role. Significant interaction effects were observed for C × H and C × H × M on AA, DHAA, and TAA (*p* < 0.05), with moderate-to-large effect sizes (partial η^2^ > 0.232), indicating that the influence of harvest timing on vitamin C accumulation is strongly dependent on cultivar and modulated by maturity stage. Although maturity alone showed a negligible main effect, its interaction with cultivar and harvest period suggests a conditional role in regulating ascorbate dynamics. These results confirm that vitamin C variability in strawberries is governed by a complex interplay of genetic and environmental factors. The observed seasonal increase in vitamin C content toward October can be attributed to environmental factors, including lower temperatures, reduced light intensity, and shorter photoperiods. Cooler conditions are known to enhance ascorbic acid stability by limiting oxidative degradation, whereas higher temperatures during early harvest months (August–September) may accelerate enzymatic oxidation and metabolic turnover of AA. Moreover, agronomic conditions such as cultivation systems, such as greenhouse versus open-field production, nutrient availability, and irrigation practices may also contribute to the observed variability in vitamin C accumulation.

**Table 1:** The partial eta-squared (η^2^) and *p*-value for main factor and its interactions by a three-way ANOVA.

	Main factors			Interactions				
	C	H	M	C*H	C*M	H*M		C* H*M
Vitamin C analysis								
Ascorbic acid	0.686***	0.784***	0.096^ns^	0.232*	0.219*	0.071^ns^		0.239*
Dehydroascorbic acid	0.456***	0.461***	0.052^ns^	0.622***	0.134^ns^	0.028^ns^		0.245*
Total ascorbic acid	0.768***	0.599***	0.005^ns^	0.557***	0.012^ns^	0.104^ns^		0.406***
Vitamin B-complex anlysis								
Thiamine (B_1_)	0.420***	0.698***	0.324***	0.246*	0.073^ns^	0.348***		0.176^ns^
Riboflavin (B_2_)	0.877***	0.443***	0.085^ns^	0.349**	0.019^ns^	0.244**		0.320**
Nicotinic acid (B_3-acid_)	0.531***	0.144^ns^	0.621***	0.083^ns^	0.428***	0.139^ns^		0.033^ns^
Niacinamide (B_3-amide_)	0.144^ns^	0.845***	0.332***	0.232*	0.136^ns^	0.239**		0.371**
D-Pantothenic acid (B_5_)	0.641***	0.022^ns^	0.889***	0.097^ns^	0.152^ns^	0.106^ns^		0.074^ns^
Pyridoxine (B_6_)	0.802***	0.483***	0.696***	0.324**	0.435***	0.036^ns^		0.266*
Biotin (B_7_)	0.428***	0.623***	0.716***	0.260*	0.120^ns^	0.193*		0.132^ns^
∑ Vitamin B	0.598***	0.040^ns^	0.873***	0.096^ns^	0.215*	0.088^ns^		0.102^ns^
Targeted phenolic compounds analysis								
Gallic acid	0.075^ns^	0.403***	0.013^ns^	0.167^ns^	0.113^ns^	0.054^ns^		0.245*
Salicylic acid	0.461***	0.479***	0.010^ns^	0.323**	0.123^ns^	0.090^ns^		0.149^ns^
*p*-Hydroxybenzoic acid	0.125^ns^	0.362***	0.755***	0.177^ns^	0.039^ns^	0.138^ns^		0.179^ns^
Gentisic acid	0.095^ns^	0.552***	0.000^ns^	0.143^ns^	0.080^ns^	0.019^ns^		0.240*
Protocatechuic acid	0.135^ns^	0.221*	0.264***	0.094^ns^	0.079^ns^	0.131^ns^		0.137^ns^
Ellagic acid	0.150^ns^	0.273**	0.001^ns^	0.155^ns^	0.026^ns^	0.038^ns^		0.205^ns^
∑ Hydroxybenzoic acid	0.097^ns^	0.319***	0.003^ns^	0.144^ns^	0.025^ns^	0.033^ns^		0.226^ns^
Ferulic acid	0.347***	0.448***	0.002^ns^	0.197^ns^	0.154*	0.026^ns^		0.213^ns^
Caffeic acid	0.226*	0.312**	0.278***	0.283*	0.085^ns^	0.014^ns^		0.237*
*trans*-Cinnamic acid	0.869***	0.213*	0.570***	0.591***	0.239**	0.042^ns^		0.329**
Chlorogenic acid	0.852***	0.064^ns^	0.277***	0.026^ns^	0.120^ns^	0.118^ns^		0.210^ns^
*p*-Coumaric acid	0.734***	0.441***	0.756***	0.129^ns^	0.746***	0.066^ns^		0.297*
∑ Hydroxycinnamic acid	0.871***	0.109^ns^	0.734***	0.375**	0.592***	0.001^ns^		0.203^ns^
∑ Phenolic acid	0.374***	0.227*	0.326***	0.214^ns^	0.216*	0.026^ns^		0.220^ns^
Quercetin	0.276**	0.600***	0.044^ns^	0.275*	0.103^ns^	0.010^ns^		0.431***
Kaempferol	0.096^ns^	0.233**	0.059^ns^	0.039^ns^	0.062^ns^	0.020^ns^		0.269*
Rutin	0.448***	0.152^ns^	0.006^ns^	0.356**	0.121^ns^	0.049^ns^		0.470***
∑ Flavonol	0.230**	0.407***	0.052^ns^	0.128^ns^	0.011^ns^	0.022^ns^		0.425***
(+)-Catechin	0.233**	0.612***	0.005^ns^	0.231*	0.010^ns^	0.038^ns^		0.028^ns^
Naringin	0.861***	0.124^ns^	0.184**	0.127^ns^	0.173*	0.039^ns^		0.222^ns^
Peonidin 3-O-ß glucoside	0.572***	0.298**	0.414***	0.648***	0.295**	0.043^ns^		0.694***
Cyanidin 3-O-ß-glucoside	0.419***	0.270**	0.759***	0.268*	0.189*	0.104^ns^		0.384**
Pelargonidin 3-O-glucoside	0.357***	0.257**	0.930***	0.793***	0.035^ns^	0.090^ns^		0.865***
∑ Anthocyanin	0.359***	0.275**	0.930***	0.787***	0.045^ns^	0.069^ns^		0.861***
∑ Flavonoid	0.356***	0.272**	0.929***	0.784***	0.045^ns^	0.064^ns^		0.860***
∑ Phenolic compounds	0.337***	0.118^ns^	0.925***	0.759***	0.001^ns^	0.035^ns^		0.834***

C: cultivar, H: harvest month, M: maturity, ∑ Vitamin B: sum of vitamin B contents, ∑ Phenolic acid: sum of phenolic acid, ∑ Flavonoid: sum of flavonoid, ∑ Phenolic compounds: sum of total phenolic compounds, ns = non-significant, * = *p* < 0.05, ** = *p* < 0.01, *** = *p* < 0.001.

**Fig. 2:**
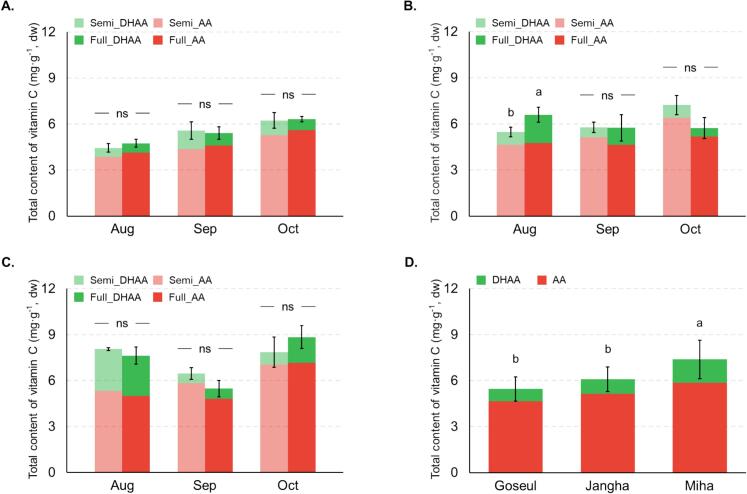
Effects of fruit maturity and harvest month on the vitamin C content (mg∙g^−1^, dw) of each strawberry cultivar. (A) Goseul, (B) Jangha, and (C) Miha. (D) Comparison of the mean vitamin C content among cultivars (mg∙g^−1^, dw). Mean values represent the average vitamin C content for each cultivar across all harvest months (August, September, and October) and maturity stages (semi and full). The error bars indicate the standard deviation (SD) of the total ascorbic acid (TAA) content, not the individual AA or DHAA components.^a-b^ Values with different superscripts differ significantly by maturity stage or cultivar (*p* < 0.05). ns, not significant; AA, ascorbic acid; DHAA, dehydroascorbic acid.

Genotype-specific responses were clearly evident, as demonstrated by the distinct behavior of the ‘Miha’ cultivar, which showed increased vitamin C levels only at the semi-ripe stage (Table S2). Such variability may arise from differences in the regulation of key enzymes involved in the L-galactose pathway and ascorbate recycling systems, leading to differential biosynthetic and degradation capacities among cultivars (Do Nascimento et al., 2005). Furthermore, variability in DHAA trends across harvest months suggests differences in redox balance and oxidative stress responses, which are also genotype-dependent. The discrepancies between the present findings and those reported by Simkova et al. (2023), who observed peak AA levels in August in ‘Capri’ strawberries followed by a decline toward November, can be explained by differences in environmental and agronomic conditions. Fruit harvested in mid-August, corresponding to longer photoperiod conditions, exhibited relatively higher levels of phytochemicals; however, these trends varied depending on cultivar and environmental context (Table S2; Fig. 2A–C). Such discrepancies may reflect differences in cultivar-specific traits, environmental conditions, or maturity classification criteria across studies. As shown in Fig. 2A–C and supported by the data presented in Table S2, TAA, AA, and DHAA levels did not differ significantly between maturity stages in most cases for all cultivars, except for the Jangha cultivar harvested in August, where maturity-dependent variation was observed. This indicates that maturity effects are limited and highly cultivar-specific. This trend is partially consistent with previous findings, in which Olsson et al. (2004) reported that vitamin C content (both reduced and oxidized forms) in the ‘Senga Sengana’ cultivar was not influenced by maturity stage, whereas the ‘Honeoye’ cultivar showed increased reduced vitamin C at full ripeness. Similarly, Cruz-Rus et al. (2011) demonstrated an increase in AA concentration during ripening from green to red stages. Overall, the present results suggest that the effect of fruit maturity on vitamin C concentration is not uniform but varies depending on cultivar, with most cultivars showing minimal or no significant changes across maturity stages (Fig. 2A–C; Table S2). Figs. 2A–C show that the vitamin C content was generally similar between semi-ripe and fully ripe fruits, with most maturity-stage comparisons being non-significant (ns). Consistent with this observation, fruit maturity had no significant effect on the TAA content (η^2^ = 0.005, *p* > 0.05; Table 1). The limited maturity effect may be attributable to the fact that both sampling stages fell within the commercially marketable ripeness window, during which vitamin C accumulation may have stabilized. Consequently, cultivar and harvest month exerted greater influence on vitamin C content than ripeness within the maturity range examined. Furthermore, when harvest month and maturity were not included as factors, the Miha cultivar exhibited a significantly higher average TAA content compared to Jangha and Goseul (*p* < 0.05), as clearly illustrated in Fig. 2D.

The cultivar-dependent differences in vitamin C content observed in this study agree with previous reports identifying genotype as a key determinant of ascorbic acid accumulation in strawberries (Cordenunsi et al., 2005; Olsson et al., 2004; Tulipani et al., 2008; Van de Velde et al., 2013). Miha showed the highest mean TAA content, followed by Jangha and Goseul (Fig. 2D), whereas ANOVA confirmed a strong cultivar effect (η^2^ = 0.768, *p* < 0.001; Table 1). Although significant, the extent of variation among the cultivars in the present study was smaller than the 2–3-fold differences reported in previous studies. This variability can be explained by differences in the regulation of key enzymes involved in the L-galactose biosynthetic pathway, including GDP-mannose pyrophosphorylase, L-galactose dehydrogenase, and L-galactono-1,4-lactone dehydrogenase, which directly control ascorbic acid synthesis. In addition, the genotype-specific efficiency of the ascorbate–glutathione cycle influences the regeneration of reduced AA from its oxidized form, DHAA, thereby affecting the overall vitamin C pool. The predominance of AA, which accounted for a maximum of 90.4% of the TAA content, compared with DHAA, which represented a maximum of 27.8% of the TAA content across cultivars, maturity stages, and harvest months (Table S2), suggests that strawberries maintain a highly reduced intracellular environment throughout fruit development. This redox balance is regulated by enzymatic antioxidants, such as dehydroascorbate reductase and glutathione reductase, which facilitate the rapid recycling of DHAA to AA. The relatively low proportion of DHAA indicates limited oxidative stress or efficient detoxification of ROS, thereby preserving vitamin C in its biologically active reduced form, as reported by Ribeiro and de Freitas (2020). Furthermore, environmental and physiological factors, including light intensity, temperature, and metabolic activity during fruit ripening, modulate the metabolism of ascorbate. Cultivation practices and nutrient availability may also regulate antioxidant metabolism by altering plant stress responses and metabolic fluxes. The high antioxidant activity observed in strawberry fruits may be explained by the accumulation of phenolic compounds, anthocyanins, and vitamin C during fruit maturation. Overall, these findings indicate that vitamin C accumulation in strawberries is governed not only by genetic factors but also by tightly regulated biochemical pathways and the maintenance of redox homeostasis. The interaction between biosynthesis, recycling, and degradation processes ultimately determines the observed variations in vitamin C content across different cultivars and environmental conditions.

### 3.2 Influence of cultivars, fruit maturity, and harvest month on variation in vitamin B-complex

Seven of the nine targeted B-complex vitamins were successfully detected and quantified in the strawberry samples (Table S4; Fig. S1), demonstrating the adequate sensitivity and linearity of the analytical method. Vitamin B9 was not quantified because the method employed was optimized for synthetic folic acid, whereas strawberries predominantly contain naturally occurring reduced folate derivatives, which require different analytical conditions for accurate detection (Siatka et al., 2025). Vitamin B12 was included in the analytical panel as part of a comprehensive B-complex profiling approach to validate its absence in plant-derived matrices and to confirm the specificity of the method. As expected, vitamin B12 was not detected in any of the strawberry samples, which is consistent with its exclusive biosynthesis by certain bacteria and archaea and its general absence in plant-based foods (Watanabe & Bito, 2018). Overall, the method exhibited strong linearity across the tested concentration ranges for all targeted vitamins (R^2^ = 0.9743–0.9997; *p* < 0.001), confirming its suitability for the quantitative analysis of B-complex vitamins in fruit matrices (Table S4). The detected vitamins (B1, B2, B3, B5, B6, and B7) were present at measurable concentrations, enabling further evaluation of their variation across harvest months, maturity stages, and cultivars. Table 1 summarizes the results of the three-way ANOVA for the B vitamins in strawberries. Cultivar (C) and maturity (M) significantly influenced the total vitamin B-complex content (*p* < 0.001), with considerable effect sizes (partial η^2^ > 0.598). A significant interaction between cultivar and maturity (C × M) was also observed (*p* < 0.05; partial η^2^ = 0.215). Vitamins B1 (thiamine), B6 (pyridoxine), and B7 (biotin) were significantly affected by cultivar, harvest month, and maturity (*p <* 0.001), each showing moderate-to-large effects (partial η^2^ > 0.324). For vitamin B5 (pantothenic acid), the most abundant B vitamin in strawberries, only cultivar and maturity were significant factors (*p* < 0.001; partial η^2^ > 0.641), indicating strong effects on its content. Conversely, the harvest month and higher-order interactions were not significant (partial η^2^ < 0.152), suggesting minimal practical impact.

**Fig. 3:**
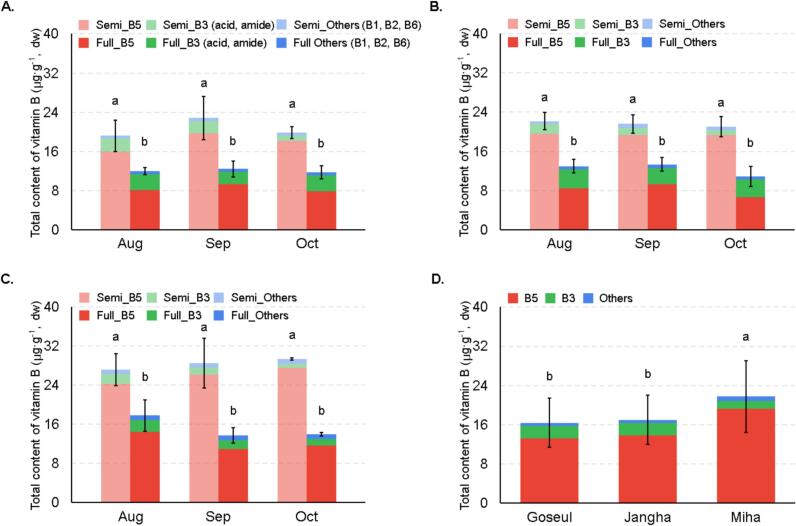
Effects of fruit maturity and harvest month on vitamin B-complex content (μg∙g^−1^, dw) in each strawberry cultivar. (A) Goseul, (B) Jangha, and (C) Miha. (D) Comparison of the mean vitamin B-complex content among cultivars (μg∙g^−1^, dw). Mean values represent the average vitamin B-complex content for each cultivar across all harvest months (August, September, and October) and maturity stages (semi and full). The error bars indicate standard deviation (SD) with respect to the total vitamin B-complex, not the individual vitamin B content.^a-b^ Values with different superscripts differ significantly by maturity stage or cultivar (*p* < 0.05). B1, thiamine; B2, riboflavin; B3-acid, nicotinic acid; B3-amide, niacinamide; B5, D-pantothenic acid; B6, pyridoxine; B7, biotin.

Table S7 presents the effects of ripeness and harvest month on the vitamin B-complex content of strawberry cultivars. The mean total vitamin B-complex content and vitamin B5 did not differ significantly among the harvest months (*p* > 0.05). In contrast, vitamin B3-amide (niacinamide) levels progressively decreased with later harvests (August > September > October; *p* < 0.05). Similarly, Hrubša et al. (2022) reported stable total B-complex and vitamin B5 contents across harvests, whereas niacinamide levels declined during later harvest periods, possibly due to increased metabolic utilization during fruit development. Comparable decreases in B-vitamin concentrations during successive harvests have also been reported for tomatoes (Kavitha et al., 2015). Figs. 3A–C and Table S7 illustrate the effects of fruit maturity on the vitamin B-complex content. In semi-ripened fruits, the total vitamin B-complex content ranged from 19.23 to 22.83 μg·g^−1^ dw across harvest months in Goseul (August: 19.23, September: 22.83, October: 19.85 μg·g^−1^ dw), 20.99–22.15 μg·g^−1^ dw in Jangha (August: 22.15, September: 21.60, October: 20.99 μg·g^−1^ dw), and 27.15–29.38 μg·g^−1^ dw in Miha (August: 27.15, September: 28.48, October: 29.38 μg·g^−1^ dw). At full ripeness, total vitamin B-complex concentrations decreased significantly to 11.74–12.47 μg·g^−1^ dw in Goseul, 10.92–13.34 μg·g^−1^ dw in Jangha, and 13.68–17.79 μg·g^−1^ dw in Miha (*p* < 0.05) (Table S7). These findings indicate that fruit maturation exerted a stronger influence on the total vitamin B-complex content than the harvest month, with substantial reductions occurring as fruits progressed from the semi-ripe to fully ripe stages. Across cultivars and harvest months, immature strawberries generally exhibited higher total vitamin B levels than fully mature fruits, although the significance of this difference varied depending on the cultivar and sampling time. In several cases, vitamin B5, the most abundant B vitamin in strawberries, also showed higher levels in immature fruits, but with cultivar- and month-specific variations. Conversely, vitamin B3 amide levels tended to be higher in mature fruits (1.33–2.01 μg g^−1^ dw), approximately 1.4-fold higher, except in Miha and Jangha harvested in October (0.64 and 1.01 μg g^−1^ dw, respectively), for which no such trend was observed. Immature strawberries have significantly higher levels of total vitamin B and B5, most likely due to active metabolic production, whereas mature fruits exhibit enhanced B3-amide formation, indicating ripening-related pathways (Duan, Wang, Tang, et al., 2025; Fecka et al., 2021). During strawberry ripening, extensive metabolic reprogramming occurs, including enhanced sugar accumulation, organic acid turnover, and shifts in primary metabolic pathways, which may influence the biosynthesis and accumulation of B vitamins (Fait et al., 2008; Perotti et al., 2023). These vitamins function as essential cofactors in central metabolic processes, such as glycolysis and amino acid metabolism, suggesting that their levels may be modulated by ripening-associated metabolic fluxes (Giampieri et al., 2012). Additionally, enzymatic activity and gene expression changes during strawberry maturation can further regulate B-vitamin content (Sánchez-Gómez et al., 2022). However, direct evidence of maturity-dependent variations in B-complex vitamins in strawberries remains limited, highlighting the need for targeted biochemical and metabolomic investigations.

When the harvest month and maturity were not factored into the analysis, the Miha cultivar exhibited an approximately 1.2-fold higher mean total vitamin B concentration than the Jangha and Goseul cultivars (*p* < 0.05; Fig. 3D). Comparable cultivar-dependent differences have been reported previously; Yoon et al. (2019) observed more than two-fold difference in vitamins B1, B2, and B3 among apple, peach, and strawberry cultivars. Similarly, Nagai et al. (2018) reported that the everbearing cultivar ‘Dekoruju’ contained more vitamin B1 but less vitamin B2 than other cultivars. Collectively, these studies indicate that the vitamin B-complex content differs significantly among strawberry cultivars. Cultivation strategies and environmental conditions have also been identified as key determinants of vitamin B-complex levels (Yang et al., 2024). Overall, these results support the notion that cultivar is a major contributor to the variation in vitamin B-complex composition. To evaluate the nutritional relevance of the observed variations in B-complex vitamins, the contribution of strawberry consumption to the recommended daily intake (RDI) was estimated based on the average vitamin concentrations and standard serving size (100 g fresh weight) (Tulipani et al., 2008). The results indicate that strawberries provide a relatively small proportion of the daily requirements for most B vitamins, typically contributing less than 5–10% of the RDI, depending on the vitamin and harvest period. Although a decreasing trend in certain B vitamins was observed in later harvest months and with increased ripeness, the significance of this variation resulted in only minor differences in RDI coverage. For instance, reductions in vitamin B1, B2, and B6 levels across harvest months translated into marginal changes in dietary contribution, suggesting a limited practical impact on overall nutritional intake. These findings indicate that while harvest timing influences the biochemical composition of strawberries, its effect on meeting daily B-vitamin requirements is minimal. Nevertheless, such variations may be relevant in the context of cumulative dietary intake or in populations with marginal nutrient deficiency.

### 3.3 Influence of cultivars, fruit maturity, and harvest month on variations in targeted phenolics

**Fig. 4:**
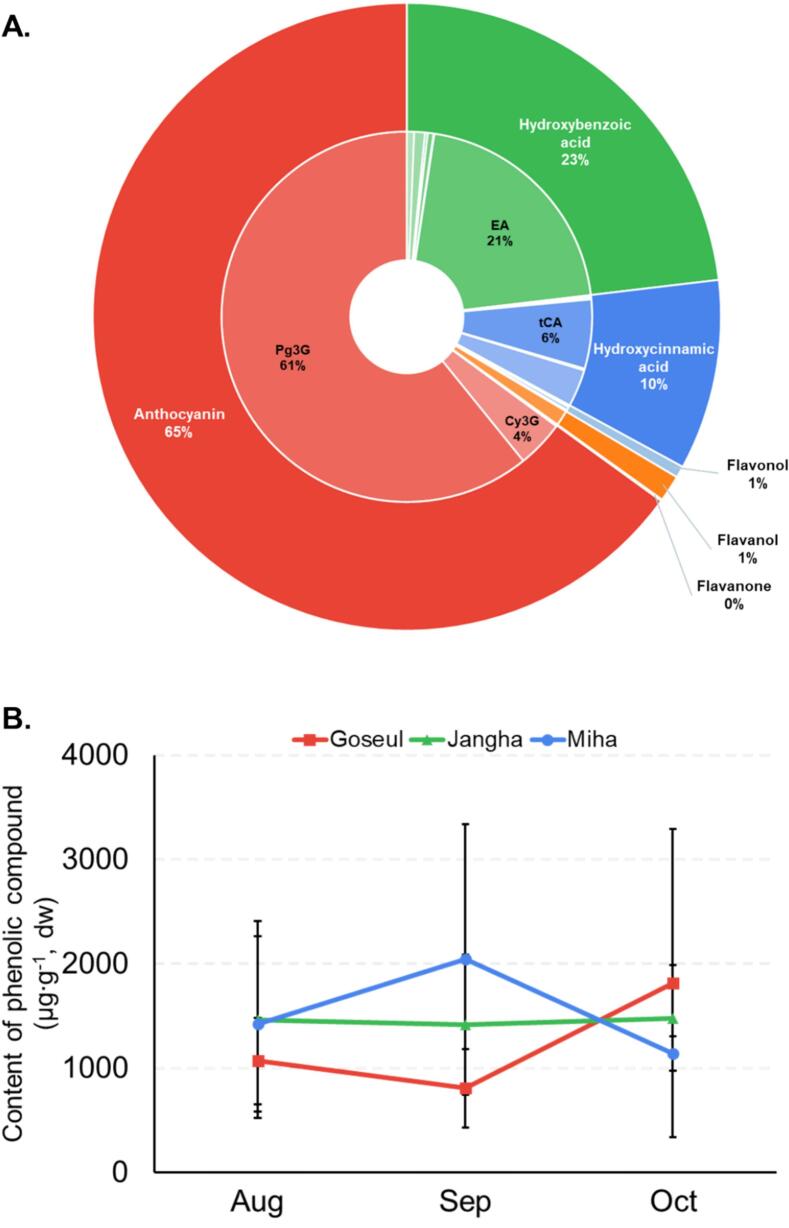
Effects of cultivar and harvest month on phenolic profiles in strawberries. (**A**) Average proportional distribution (%) of phenolic compounds. The average value represents the distribution of phenolic compounds across strawberries, including cultivar (Goseul, Jangha, and Miha), harvest months (August, September, and October), and maturity stages (semi and full). The inner ring represents identified phenolic compounds, while the outer ring indicates their subclasses. EA, ellagic acid; tCA, trans-cinnamic acid; Cy3G, cyanidin 3-O-ß-glucoside; Pg3G, pelargonidin 3-O-glucoside. (**B)** Comparison of phenolic compound contents (μg∙g^−1^, dw) in each cultivar by harvest month. The mean values represent averages across phenolic compound distribution in strawberries, including maturity stages (semi and full).

Phenolic compounds contribute to antioxidant defense through mechanisms such as free radical scavenging, metal ion chelation, and inhibition of lipid peroxidation, thereby reducing oxidative damage in biological systems (Duan, Bao, Jiang, et al., 2025; Duan, Wang, Song, et al., 2025). In addition to their antioxidant properties, strawberry anthocyanins and other phenolic compounds have been reported to exhibit anti-inflammatory, antimicrobial, cardioprotective, and anticancer activities by modulating oxidative stress and inflammatory signaling pathways (Duan, Wang, Song, et al., 2025; Giampieri et al., 2012). Among the 59 phenolics studied, 19 were detected in strawberries, including 11 phenolic acids and 8 flavonoids (Table S6 and Fig. S2). Across cultivars, maturation stages, and harvest months, anthocyanins were the most abundant category, accounting for an average of 65% of the total phenolic content, followed by hydroxybenzoic (23%) and hydroxycinnamic acids (10%). The most abundant phytochemical compound was pelargonidin 3-O-glucoside (61%), followed by ellagic acid, trans-cinnamic acid, and cyanidin 3-O-β-glucoside (Fig. 4A). Among these metabolites, anthocyanins, particularly pelargonidin- and cyanidin-based derivatives, are considered major contributors to both fruit color and antioxidant capacity. Increased anthocyanin biosynthesis during ripening is closely associated with the development of red pigmentation and enhanced antioxidant potential in fully matured fruits (Martínez-Ferri et al., 2023). This phenolic profile is consistent with that of previous studies, indicating that strawberries are rich in anthocyanins, with pelargonidin 3-O-glucoside being the predominant compound (Martínez-Ferri et al., 2023). However, the relatively high hydroxybenzoic acid content observed in this study contrasts with that reported for certain cultivars (Atazhanova et al., 2025; Duarte et al., 2018). These discrepancies may be attributed to several factors, including cultivar-specific metabolic variations, environmental conditions (such as temperature, light intensity, and soil composition), and differences in harvest timing and fruit maturation. Notably, the three-way ANOVA results (Table 1) demonstrated that the harvest month (H) and maturity stage (M) significantly influenced *p*-hydroxybenzoic acid levels (*p* < 0.001), suggesting that temporal and developmental factors play critical roles in their accumulation. Additionally, variations in extraction protocols and analytical sensitivity across studies may have further contributed to these differences in results. Therefore, the elevated hydroxybenzoic acid levels observed in this study likely reflect the combined effects of genotype, environmental conditions, and postharvest biochemical dynamics. The phenolic content differed considerably by cultivar and maturity (*p* < 0.001), with substantial effect sizes (partial η^2^ = 0.337 and 0.925, respectively), whereas harvest month was not significant (partial η^2^ = 0.118). Significant interactions between cultivar × harvest month (C × H) (*p* < 0.001; partial η^2^ = 0.759) and cultivar × harvest month × maturity (C × H × M) (*p* < 0.001; partial η^2^ = 0.834) were detected, indicating that the effect of C × H varied with maturity. Flavonoid and phenolic acid contents were significantly influenced by each main factor (C, H, M) (*p* < 0.05; partial η^2^ > 0.227), with flavonoids showing a particularly strong effect of maturity (partial η^2^ = 0.929). The C × H and C × H × M interactions had considerable effects on flavonoid content (partial η^2^ > 0.784), whereas phenolic acids were not affected, suggesting that flavonoid accumulation is more sensitive to cultivar–harvest month interactions and maturity than to phenolic acid accumulation. Among the individual metabolites, all anthocyanins (peonidin 3-O-β-glucoside, cyanidin 3-O-β-glucoside, and pelargonidin 3-O-glucoside) differed significantly across cultivars, harvest months, and maturity (*p* < 0.01), as well as for C × H × M interactions. In contrast, the content of ellagic acid, the second most abundant phenolic acid, varied only with the harvest month (*p* < 0.01). No significant differences were observed in the harvest month × maturity interaction (H × M) for any of the detected metabolites. Overall, strawberry phenolics revealed significant cultivar- and maturity-dependent variation, consistent with studies showing that genotype and ripeness dominate phenolic production, whereas the harvest month contributes less (Kosar et al., 2004). The strong effects of C × H × M on flavonoids are consistent with previous studies on the environmental modification of anthocyanin. However, the modest response to ellagic acid indicates its compound-specific stability.

Tables S8–S11 and Fig. 4B show the effects of harvest month on the phenolic metabolite content of the strawberry cultivars. The total phenolic content did not exhibit a uniform response to the harvest month; instead, the temporal pattern varied markedly among cultivars (Fig. 4B). This cultivar-specific response was supported by the highly significant cultivar × harvest month interaction observed for total phenolic compounds (C × H: η^2^ = 0.759, *p* < 0.001; Table 1), indicating that the influence of harvest timing strongly depended on the genotype. Specifically, Goseul and Miha displayed contrasting seasonal trends from August to October, whereas Jangha exhibited a comparatively different response pattern. Goseul exhibited relatively low phenolic content during the early harvest months (August and September), but increased substantially in October, whereas Miha showed the opposite trend across the same period (Fig. 4B). Consequently, the ranking of cultivars based on phenolic content changed throughout the harvest season, demonstrating that the harvest month alone was not a reliable predictor of phenolic accumulation. The distinct responses among cultivars may be attributed to differences in their genetic backgrounds and physiological adaptations to changing environmental conditions during the production season. The day-neutral cultivar Goseul and the everbearing cultivars Jangha and Miha differ in flowering and fruiting characteristics, which may influence the biosynthesis and accumulation of phenolic compounds under varying seasonal conditions. These findings emphasize that phenolic accumulation in strawberries is governed by the interaction between genotype and harvest period, rather than by either factor independently. Notably, a harvest break after September allows for recovery from high-temperature stress or gradual depletion of plant vigor (Lee et al., 2017; Ra et al., 1996). Overall, harvest-month effects differed by cultivar; everbearing varieties exhibited varying seasonal sensitivity, whereas the day-neutral Goseul accumulated more phenolics later in the season, indicating genotype-specific responses to temperature and light changes (Ruan et al., 2013). Previous studies have shown that strawberry phenolic accumulation is temperature-dependent; higher growing temperatures promote total anthocyanin, flavonoid, and phenolic contents, whereas lower temperatures inhibit or delay antioxidant synthesis (Wang & Zheng, 2001). Similarly, anthocyanin accumulation increases at higher daytime and nighttime temperatures (Cordenunsi et al., 2005). In this study, the average temperatures in the Daegwallyeong region decreased from 21.4 °C in August to 17.0 °C in September and 9.0 °C in October, which likely contributed to the observed decline in average phenolic content in the later harvest months.

**Fig. 5:**
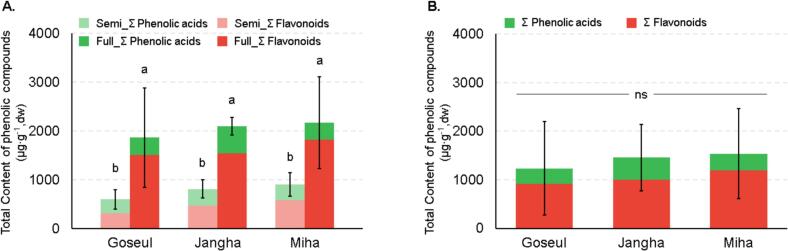
Effects of cultivar, fruit maturity, and harvest month on the phenolic content of strawberries. (**A**) Mean phenolic compound content by maturity stage and cultivar (μg∙g^−1^, dw). The error bars indicate standard deviation (SD) for the sum of phenolic compounds.^a-b^ Values with different superscripts differ significantly by maturity stage within strawberry cultivars (*p* < 0.05). (**B**) Phenolic compound contents depending on cultivars (μg∙g^−1^, dw). The mean value represents the average phenolic content in strawberries by the cultivar across all harvest months (August, September, and October) and maturity stages (semi and full). The error bars indicate the standard deviation (SD) with respect to the total phenolic content, not each sub phenolic group content. ns, not significant.

Fig. 5A shows the effect of fruit maturity on the phenolic content of strawberries. An overall increase in total phenolics and flavonoids was observed with ripening; however, the magnitude of change varied among cultivars, with fold-increases ranging from approximately 2.0- to 3.0-fold, depending on the genotype (Fig. 5A). This increase was primarily attributed to pelargonidin 3-O-glucoside, which was up to 3.2-fold more abundant in mature fruits. Consistent with previous studies, anthocyanin content increased significantly during strawberry ripening, reflecting enhanced pigment biosynthesis and fruit maturation (Aaby et al., 2012). Conversely, the ellagic acid concentrations did not change significantly with maturity (*p* > 0.05), consistent with the findings for the Senga Sengana cultivar (Olsson et al., 2004). However, previous studies have reported inconsistent trends, with some indicating an increase (Williner et al., 2003) and others indicating a decrease (Abe et al., 2012) in ellagic acid levels during ripening. These discrepancies may be attributed to differences in cultivar genetics, environmental growing conditions, and analytical approaches. In addition, ellagic acid predominantly exists as ellagitannins, which are synthesized during early fruit development and may undergo hydrolysis, degradation, or conversion to other phenolic derivatives during fruit ripening. Variations in enzyme activity (e.g., tannase and polyphenol oxidase) and metabolic flux through the phenylpropanoid pathway may further contribute to inconsistencies observed across studies. In this study, no significant differences were observed in the phenolic content among the cultivars (Fig. 5B). In contrast, previous studies have reported cultivar-dependent variations, with phenolic content differing between up to 2.3-fold among 27 genotypes (Aaby et al., 2012) and 1.8-fold across nine genotypes (Tulipani et al., 2008). The lack of significant variation in the present study may be attributed to the similarities in the ecotypes of the selected cultivars and controlled cultivation conditions.

### 3.4 Principal component analysis

**Fig. 6:**
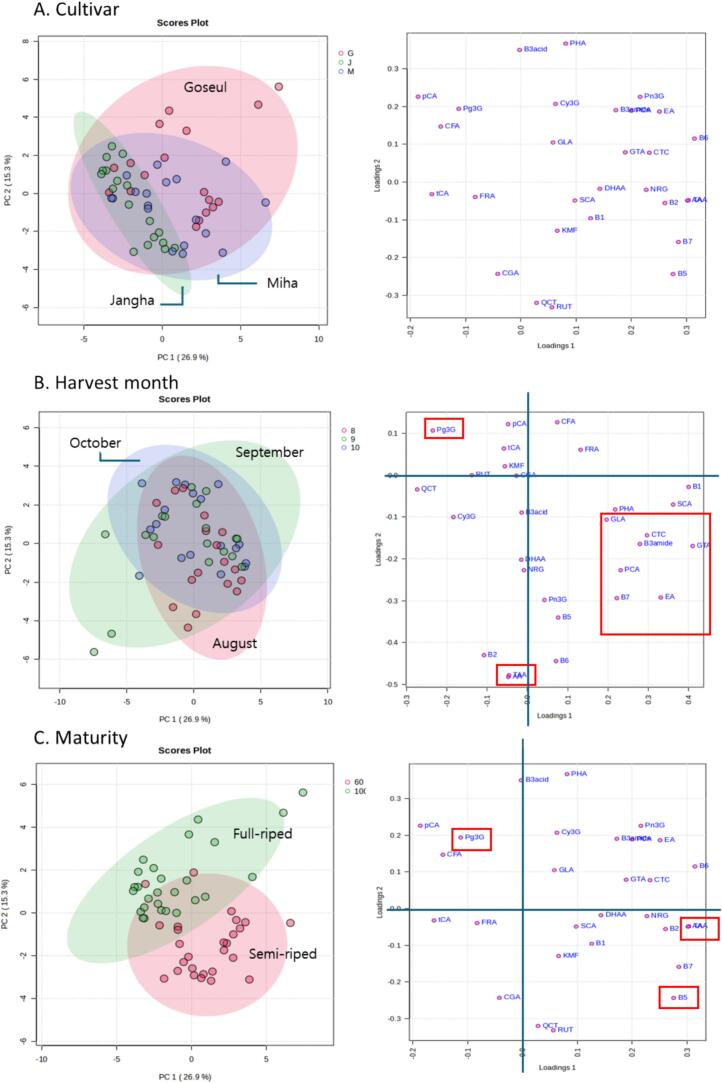
Principal component analysis (PCA) of strawberries by cultivar, harvest month, and maturity stage. (A) Score (left) and loading (right) plots by cultivar. (B) Score (left) and loading (right) plots by harvest months. (C) Score (left) and loading (right) plots by maturity stage.

PCA is a multivariate statistical method used to reduce high-dimensional data and determine the correlations between associated variables (Kim & Kim, 2012). In this study, PCA was performed on 29 metabolites quantified in strawberries across cultivars, harvest months, and maturation stages (Fig. 6). The score plot represents the sample variance, whereas the loading plot indicates the contribution of each variable to the PC1 and PC2. PC1 and PC2 explained 26.9% and 15.3% of the total variance, respectively, accounting for a cumulative 42.2% of the overall variance. This relatively low variance, together with the absence of clear group separation, indicates the limited discriminatory power of PCA, suggesting the observed clustering patterns of the data points. The PCA distribution by cultivar (Fig. 6A) showed overlapping patterns between the day-neutral Goseul and the everbearing Jangha and Miha cultivars. Goseul was mainly distributed in the first quadrant, whereas Jangha and Miha were primarily located in the second and fourth quadrants, respectively. This indicates that day-neutral and everbearing cultivars share similar metabolite profiles, with day-neutral cultivars exhibiting traits characteristic of the everbearing cultivars. Additionally, the two everbearing cultivars displayed closely aligned genetic and physiological patterns, suggesting minimal metabolic divergence.

The PCA distribution by harvest month (Fig. 6B) showed a substantial overlap among the samples. Metabolites that decreased with the harvest month, such as B vitamins and hydroxybenzoic acid, were positioned in the fourth quadrant, whereas those that increased, such as vitamin C and pelargonidin 3-O-glucoside, were located in the second and third quadrants, respectively. In contrast, maturity (Fig. 6C) clearly separated the samples; as fruits progressed from immature to mature, their distribution shifted from the fourth to the second quadrant along PC1 and PC2. Notably, pelargonidin 3-O-glucoside, whose content increased with ripening, was associated with the second quadrant, whereas vitamins B5 and C, whose levels decreased with maturity, were located in the fourth quadrant. In immature fruits, pelargonidin 3-O-glucoside was moderately positively correlated with vitamin B5 and TAA (*r* > 0.4; *p* < 0.05). However, this correlation was not significant in mature fruits (*r* < −0.1, not significant), indicating a significant impact of maturity on metabolic relationships (Figs. S3 and S4). The PCA overlap across harvest months suggests minor month-driven metabolic alterations, consistent with previous studies indicating that ecological month-to-month variations have minimal effects on strawberry metabolic profiles (Adrian et al., 2024). In contrast, the pronounced maturity-based separation is consistent with the results of previous studies that reported that ripening is the primary source of phenolic and vitamin dynamics (Coyago-Cruz et al., 2025). The altered relationships in mature fruits further support prior observations of maturity-specific metabolic reprogramming (Bian et al., 2025).

## 4 Conclusion

This study systematically evaluated the variation in functional nutritional metabolites of everbearing (Jangha and Miha) and day-neutral (Goseul) strawberries cultivated under highland summer conditions with respect to cultivar, harvest timing, and fruit maturity. The findings identified fruit maturity, within the three cultivars and harvest months (seasons) examined, as the predominant factor influencing metabolite composition, particularly for phenolic compounds and B-complex vitamins. The effect of harvest month was comparatively variable and less pronounced. Distinct cultivar-dependent patterns were also observed, with Miha exhibiting relatively higher vitamin content and Goseul showing enhanced phenolic accumulation during the later harvest period. From an applied perspective, these results provide valuable insights for optimizing harvest strategies and cultivar selection to achieve targeted nutritional profiles, thereby potentially enhancing product marketability and improving consumer health. However, this study has several limitations, including the evaluation of only three cultivars within a single growing season and the lack of detailed environmental monitoring, both of which may contribute to variability in the metabolite levels. Furthermore, the inability to detect specific compounds such as folate highlights the need for more sensitive analytical approaches. Future research incorporating a broader range of cultivars, multi-seasonal evaluations, and comprehensive environmental characterizations is essential to validate and expand these findings. Overall, this study provides a useful framework for improving strawberry crop management practices aimed at enhancing the functional nutritional quality of strawberries grown in highland cultivation systems.

## CRediT authorship contribution statement

**Ji-Ye Kim:** Writing – original draft, Formal analysis. **Muthu Thiruvengadam:** Writing – original draft. **Doyeon Kim:** Methodology, Formal analysis. **Hee-Youn Chi:** Methodology, Data curation. **Hee-Jin Choi:** Project administration, Methodology. **Ja-Min Lee:** Software, Resources. **Dagyeom Jeon:** Visualization, Resources. **Yunwoo Park:** Methodology, Formal analysis. **Seung-Hyun Kim:** Writing – review & editing, Validation, Supervision, Investigation, Funding acquisition, Conceptualization.

## Ethics statement

Ethical approval was not applicable for this study.

## Funding

This work was supported by the National Research Foundation of Korea (NRF) grant funded by the Korea government (MSIT) (RS-2025-00557207). This study was also funded by Rural Development Administration (grant number PJ01677502) in Republic of Korea.

## Declaration of competing interest

The authors declare that they have no known competing financial interests or personal relationships that could have appeared to influence the work reported in this paper.

## Data Availability

Data will be made available on request.
