# Supplementary material for: Influence of cultivar, fruit maturity, and harvest month on the targeted metabolite profile of Highland strawberries (Fragaria × ananassa Duch.)
Source: Food Chem X. 2026 Jun 6;37:104077. doi: 10.1016/j.fochx.2026.104077 (PMC13276600; doi:10.1016/j.fochx.2026.104077)
Supplement: Supplementary file 1 — Supplementary material [file mmc1.docx]

**[Supplementary Materials] including 11 Tables and 4 Figures**

**Influence of Cultivar, Fruit Maturity, and Harvest Month on the Targeted Metabolite Profile of Highland Strawberries (*Fragaria* × *ananassa* Duch.)**

**Ji-Ye Kim^a,†^, Muthu Thiruvengadam^a,†^, Doyeon Kim^b^, Hee-Youn Chi^a^, Hee-Jin Choi^a^, Ja-Min Lee^a^, Dagyeom Jeon^a^, Yunwoo Park^a^, Seung-Hyun Kim^a,*^**

*^a^ Department of Crop Science, College of Life Science, Konkuk University, Seoul 05029, Republic of Korea*

^b^ Highland Agriculture Research Institute, National Institute of Crop Science, Rural Development Administration, Pyeongchang 25342, Korea

^†^ These authors equally contributed to this work.

*** Corresponding author.** Prof. Seung-Hyun Kim

**E-mail address:** [kshkim@konkuk.ac.kr](mailto:kshkim@konkuk.ac.kr); Tel: +82-02-2049-6163;

**Table S1**. A monthly temperature and relative humidity (%RH) information in a rain shelter house during the entire cultivation period (April to November 2023) of ever-bearing strawberries in Pyeongchang, Korea.

|  |  | Temperature, °C | | |  | Relative humidity, % |
| --- | --- | --- | --- | --- | --- | --- |
|  |  | Mean | Max. | Min. |  |  |
| April 27 – 30 |  | 17.0 | 29.0 | 9.7 |  | 54.7 |
| May 01 – 31 |  | 19.7 | 34.9 | 8.2 |  | 61.5 |
| June 01 – 30 |  | 20.8 | 41.3 | 12.7 |  | 71.5 |
| July 01 – 31 |  | 23.8 | 36.2 | 13.8 |  | 79.1 |
| August 01 – 31 |  | 23.1 | 33.9 | 15.9 |  | 80.8 |
| September 01 – 30 |  | 20.7 | 36.8 | 10.7 |  | 79.1 |
| October 01 – 31 |  | 13.2 | 22.9 | 4.8 |  | 78.6 |

**Table S2.** Composition and content of vitamin C (mg∙g^-1^, dry weight basis, dw) in strawberries.

| Cultivar | Compound | Semi | | |  | Full | | |
| --- | --- | --- | --- | --- | --- | --- | --- | --- |
|  |  | Aug | Sep | Oct |  | Aug | Sep | Oct |
| Goseul |  |  |  |  |  |  |  |  |
|  | Ascorbic acid†  (% AA) | 3.9 ± 0.1^b^  (86.9 %) | 4.4 ± 0.4^b^  (78.4 %) | 5.3 ± 0.2^a^  (84.4 %) |  | 4.1 ± 0.2^b^  (87.3 %) | 4.6 ± 0.5^b^  (84.9 %) | 5.6 ± 0.5^a^  (88.6 %) |
|  | Dehydroascorbic acid  (% DHAA) | 0.6 ± 0.2^ns^ (13.1 %) | 1.2 ± 0.4 (21.6 %) | 1.0 ± 0.4 (15.6 %) |  | 0.6 ± 0.1^ns^  (12.7 %) | 0.8 ± 0.1 (15.1 %) | 0.7 ± 0.4 (11.4 %) |
|  | Total ascorbic acid | 4.4 ± 0.3^b^ | 5.6 ± 0.6^a^ | 6.2 ± 0.5^a^ |  | 4.7 ± 0.3^c^ | 5.4 ± 0.4^b^ | 6.3 ± 0.2^a^ |
|  |  |  |  |  |  |  |  |  |
| Jangha |  |  |  |  |  |  |  |  |
|  | Ascorbic acid  (% AA) | 4.6 ± 0.2^c^  (85.0 %) | 5.1 ± 0.3^b^  (88.8 %) | 6.4 ± 0.1a  (88.6 %) |  | 4.8 ± 0.2^ns^  (72.2 %) | 4.6 ± 0.5 (80.8 %) | 5.2 ± 0.6 (90.0 %) |
|  | Dehydroascorbic acid  (% DHAA) | 0.8 ± 0.2^ns^  (15.0 %) | 0.6 ± 0.1 (11.2 %) | 0.8 ± 0.5 (11.4 %) |  | 1.8 ± 0.6^a^  (27.8 %) | 1.1 ± 0.7^ab^ (19.2 %) | 0.6 ± 0.1^b^  (10.0 %) |
|  | Total ascorbic acid | 5.5 ± 0.3^b^ | 5.8 ± 0.3^b^ | 7.2 ± 0.6^a^ |  | 6.6 ± 0.5^ns^ | 5.7 ± 0.9 | 5.7 ± 0.7 |
|  |  |  |  |  |  |  |  |  |
| Miha |  |  |  |  |  |  |  |  |
|  | Ascorbic acid  (% AA) | 5.3 ± 0.2^b^  (90.4 %) | 5.8 ± 0.4^b^  (90.4 %) | 7.0 ± 0.9^a^  (89.6 %) |  | 5.0 ± 0.3^b^  (87.7 %) | 4.8 ± 0.6^b^  (87.7 %) | 7.2 ± 0.2^a^  (81.1 %) |
|  | Dehydroascorbic acid  (% DHAA) | 2.7 ± 0.3^a^  (9.6 %) | 0.6 ± 0.0^b^  (9.6 %) | 0.8 ± 0.1^b^  (10.4 %) |  | 2.6 ± 0.8^a^  (12.3 %) | 0.7 ± 0.1^b^  (12.3 %) | 1.7 ± 0.7^ab^  (18.9 %) |
|  | Total ascorbic acid | 8.1 ± 0.1^a^ | 6.5 ± 0.4^b^ | 7.9 ± 1.0^a^ |  | 7.6 ± 0.6^a^ | 5.5 ± 0.5^b^ | 8.8 ± 0.7^a^ |

^a-c^ Values with different superscripts are significantly different with harvest month in maturity (*p* < 0.05). % AA = respective proportion of ascorbic acid within total ascorbic acid content, % DHAA = respective proportion of dehydroascorbic acid within total ascorbic acid content, ns = non-significant.

† Based on the calibration curve used in this study, a limit of detection (LOD) and limit of quantification (LOQ) of ascorbic acid (AA) were calculated as 1.43 ppm (μg∙mL^-1^) and 4.32 ppm (μg∙mL^-1^), respectively. The LOD and LOQ were calculated as following: LOD = 3.3ⅹ(SD of intercept/slope), LOQ = 10ⅹ(SD of intercept/slope). The instrumental analysis repeatability (n=2) within the calibration curve range (5 – 40 μg∙mL^-1^) was shown in 0.3%.

**Table S3.** Optimized MRM parameters of vitamin B-complex for LC-MS/MS analysis.

| Compound | Formula |  |  | Q1 | | Q3 | | Q3 | |
| --- | --- | --- | --- | --- | --- | --- | --- | --- | --- |
|  |  | t_R_ | Ionization | (molecular ion) | | (Quantifier ion) | | (Qualifier ion) | |
|  |  | (min) | mode | m/z | pre bias | m/z | CE | m/z | CE |
|  |  |  |  |  | (volt) |  | (volt) |  | (volt) |
| Thiamine hydrochloride (B_1_) | C_12_H_17_N_4_OS·HCl | 1.103 | + | 264.95 | -13.0 | 122.05 | -15.0 | 144.10 | -15.0 |
| Riboflavin (B_2_) | C_17_H_20_N₄O_6_ | 5.461 | + | 377.00 | -11.0 | 243.15 | -23.0 | 172.10 | -37.0 |
| Nicotinic acid (B_3-acid_) | C_6_H_5_NO_2_ | 1.916 | + | 123.10 | -14.0 | 80.10 | -21.0 | 78.10 | -24.0 |
| Niacinamide (B_3-amide_) | C_6_H_6_N₂O | 3.259 | + | 124.10 | -13.0 | 78.10 | -22.0 | 80.10 | -21.0 |
| D-Pantothenic acid hemicalcium salt (B_5_) | C_9_H_17_NO_5_·1/2Ca | 4.406 | + | 219.95 | -11.0 | 90.05 | -13.0 | 202.15 | -12.0 |
| Pyridoxine (B_6_) | C_8_H_11_NO_3_ | 2.525 | + | 169.95 | -19.0 | 134.10 | -21.0 | 152.05 | -15.0 |
| Biotin (B_7_) | C_10_H_16_N_2_O_3_S | 5.291 | + | 245.10 | -12.0 | 227.05 | -14.0 | 97.05 | -31.0 |
| Folic acid (B_9_) | C_10_H_16_N_2_O_3_S | 4.725 | + | 441.95 | -13.0 | 295.15 | -17.0 | 176.15 | -40.0 |
| Cyanocobalamin (B_12_) | C_63_H_88_CoN_14_O_14_P | 4.579 | + | 678.55 | -20.0 | 147.10 | -40.0 | 359.15 | -24.0 |

t_R_ = retention time, CE = collision energy.

**Table S4**. Calibration curves of each vitamin B-complex examined in this study.

| Compound | Regression | Linearity Range | LOD / LOQ † | Repeatability* | R^2^ | *p*-value |
| --- | --- | --- | --- | --- | --- | --- |
|  | Equation | (ng∙mL^-1^) | (ng∙mL^-1^) | (% RSD, n=2) |  |  |
| Thiamine (B_1_) | y = 245610.1x - 5992.8 | 0.05 - 2 | 0.80 / 2.43 | 3.0 | 0.9743 | *p <* 0.001 |
| Riboflavin (B_2_) | y = 16894x - 7397.7 | 1 - 7.5 | 2.65 / 8.03 | 2.2 | 0.9870 | *p <* 0.001 |
| Nicotinic acid (B_3-acid_) | y = 27258x + 23989 | 1 - 40 | 4.48 / 13.57 | 1.7 | 0.9983 | *p <* 0.001 |
| Niacinamide (B_3-amide_) | y = 121963x + 69625 | 2.5 - 25 | 1.43 / 4.34 | 0.7 | 0.9995 | *p <* 0.001 |
| D-Pantothenic acid (B_5_) | y = 10466x - 163872 | 50 - 500 | 48.67 / 147.49 | 0.6 | 0.9986 | *p <* 0.001 |
| Pyridoxine (B_6_) | y = 375899x - 842.12 | 0.5 - 5 | 0.22 / 0.67 | 0.8 | 0.9997 | *p <* 0.001 |
| Biotin (B_7_) | y = 38135x + 21166 | 0.1 - 2 | 0.12 / 0.38 | 3.6 | 0.9994 | *p <* 0.001 |

R^2^ = coefficient of determination, *p*-value = statistical significance of the slope.

† Based on the calibration curve used in this study, the limit of detection (LOD) and limit of quantification (LOQ) of vitamin B complex were calculated as following: LOD = 3.3ⅹ(SD of intercept/slope), LOQ = 10ⅹ(SD of intercept/slope).

* The repeatability meant the mean %RSD for duplicate instrumental analyses (n=2) of vitamin B complex standards used in each calibration curve range.

**Table S5**. Optimized MRM parameters of 59 targeted phenolic compounds for LC-MS/MS analysis.

| Class | Subclass | Compound | Formula |  |  |  | Q1 | | Q3 | |
| --- | --- | --- | --- | --- | --- | --- | --- | --- | --- | --- |
|  |  |  |  | t_R_ |  | Ionization | (molecular ion) | | (quantifier ion) | |
|  |  |  |  | (min) |  | mode | m/z | pre bias | m/z | CE |
|  |  |  |  |  |  |  |  | (volt) |  | (volt) |
| Phenolic acid | Hydroxybenzoic acid | 5-Sulfosalicylic acid | C₇H₆O₆S | 1.27 |  | - | 217.05 | 15.0 | 198.95 | 13.0 |
|  |  | Gallic acid | C₇H₆O₅ | 1.37 |  | - | 169.05 | 18.0 | 124.90 | 18.0 |
|  |  | Protocatechuic acid | C₇H₆O₄ | 2.24 |  | - | 153.25 | 30.0 | 109.10 | 15.0 |
|  |  | *p*-Hydroxybenzoic acid | C₇H₆O₃ | 3.00 |  | - | 137.10 | 27.0 | 93.00 | 17.0 |
|  |  | Gentisic acid | C₇H₆O₄ | 3.01 |  | - | 153.05 | 30.0 | 108.10 | 22.0 |
|  |  | Vanillic acid | C₈H₈O₄ | 3.33 |  | - | 167.05 | 11.0 | 151.95 | 16.0 |
|  |  | Syringic acid | C₉H₁₀O₅ | 3.39 |  | - | 197.10 | 21.0 | 181.85 | 16.0 |
|  |  | 3-Hydroxybenzoic acid | C₇H₆O₃ | 3.43 |  | - | 137.20 | 27.0 | 92.95 | 13.0 |
|  |  | 2,4-Dihydroxybenzoic acid | C₇H₆O₄ | 3.49 |  | - | 153.05 | 30.0 | 109.10 | 16.0 |
|  |  | Ellagic acid | C₁₄H₆O₈ | 4.20 |  | - | 301.15 | 21.0 | 145.00 | 37.0 |
|  |  | 3,4-Dimethoxybenzoic acid | C₉H₁₀O₄ | 4.55 |  | - | 181.10 | 20.0 | 137.10 | 15.0 |
|  |  | Salicylic acid | C₇H₆O₃ | 5.40 |  | - | 137.25 | 27.0 | 92.95 | 18.0 |
|  | Hydroxycinnamic acid | Chlorogenic acid | C₁₆H₁₈O₉ | 2.83 |  | - | 353.25 | 25.0 | 190.90 | 20.0 |
|  |  | Caffeic acid | C₉H₈O₄ | 3.21 |  | - | 179.10 | 19.0 | 134.90 | 16.0 |
|  |  | *p*-Coumaric acid | C₉H₈O₃ | 4.06 |  | - | 163.10 | 18.0 | 118.95 | 16.0 |
|  |  | Ferulic acid | C₁₀H₁₀O₄ | 4.37 |  | - | 193.30 | 21.0 | 134.10 | 18.0 |
|  |  | *m*-Coumaric acid | C₉H₈O₃ | 4.51 |  | - | 163.10 | 30.0 | 118.95 | 16.0 |
|  |  | *o*-Coumaric acid | C₉H₈O₃ | 5.00 |  | - | 163.10 | 18.0 | 119.10 | 14.0 |
|  |  | *trans*-Cinnamic acid | C₉H₈O₂ | 6.35 |  | + | 148.80 | -11.0 | 103.05 | -20.0 |
|  | Phenylacetic acid | Homogentisic acid | C₈H₈O₄ | 1.78 |  | - | 167.10 | 18.0 | 123.00 | 13.0 |
| Stilbenoid | Stilbene | Polydatin | C₂₀H₂₂O₈ | 4.02 |  | - | 389.15 | 28.0 | 226.90 | 16.0 |
|  |  | *trans*-Resveratrol | C₁₄H₁₂O₃ | 5.45 |  | - | 227.25 | 16.0 | 143.10 | 27.0 |
|  |  | *cis*-Resveratrol | C₁₄H₁₂O₃ | 6.00 |  | - | 227.25 | 16.0 | 185.00 | 19.0 |
| Others | Benzaldehyde | Vanillin | C₈H₈O₃ | 4.16 |  | - | 151.05 | 29.0 | 135.95 | 18.0 |
| Flavonoid | Flavonol | Rutin | C₂₇H₃₀O₁₆ | 3.83 |  | - | 609.35 | 22.0 | 299.90 | 36.0 |
|  |  | Myricetin | C₁₅H₁₀O₈ | 4.96 |  | - | 317.20 | 22.0 | 151.00 | 24.0 |
|  |  | Quercetin | C₁₅H₁₀O₇ | 5.89 |  | - | 301.20 | 21.0 | 150.90 | 22.0 |
|  |  | Kaempferol | C₁₅H₁₀O₆ | 6.77 |  | - | 285.20 | 21.0 | 117.00 | 41.0 |
|  | Flavone | Isoorientin | C₂₁H₂₀O₁₁ | 3.43 |  | - | 447.25 | 16.0 | 326.95 | 23.0 |
|  |  | Orientin | C₂₁H₂₀O₁₁ | 3.71 |  | - | 447.25 | 16.0 | 326.90 | 21.0 |
|  |  | Isovitexin | C₂₁H₂₀O₁₀ | 4.04 |  | - | 431.30 | 16.0 | 310.90 | 24.0 |
|  |  | Vitexin | C₂₁H₂₀O₁₀ | 4.10 |  | - | 431.10 | 16.0 | 310.90 | 22.0 |
|  |  | Tricetin | C₁₅H₁₀O₇ | 5.08 |  | - | 301.10 | 22.0 | 148.90 | 31.0 |
|  |  | Luteolin | C₁₅H₁₀O₆ | 5.84 |  | - | 285.20 | 20.0 | 132.90 | 35.0 |
|  |  | Apigenin | C₁₅H₁₀O₅ | 6.61 |  | - | 269.25 | 19.0 | 117.15 | 36.0 |
|  |  | Tricin | C₁₇H₁₄O₇ | 6.75 |  | - | 329.25 | 23.0 | 298.85 | 25.0 |
|  | Flavanol | (+)-Catechin | C₁₅H₁₄O₆ | 2.72 |  | - | 289.30 | 20.0 | 245.10 | 14.0 |
|  | Flavanone | Naringin | C₂₇H₃₂O₁₄ | 4.41 |  | - | 579.30 | 22.0 | 270.90 | 34.0 |
|  |  | Eriodictyol | C₁₅H₁₂O₆ | 5.74 |  | - | 287.15 | 21.0 | 150.95 | 15.0 |
|  |  | Naringenin | C₁₅H₁₂O₅ | 6.60 |  | - | 271.10 | 19.0 | 119.10 | 25.0 |
|  |  | Hesperetin | C₁₆H₁₄O₆ | 6.90 |  | - | 301.10 | 21.0 | 163.85 | 24.0 |
|  | Isoflavone | Daidzin | C₂₁H₂₀O₉ | 3.51 |  | - | 415.30 | 29.0 | 251.90 | 29.0 |
|  |  | Glycitin | C₂₂H₂₂O₁₀ | 3.59 |  | - | 445.30 | 16.0 | 282.90 | 19.0 |
|  |  | Genistin | C₂₁H₂₀O₁₀ | 4.26 |  | - | 431.25 | 30.0 | 267.90 | 28.0 |
|  |  | Daidzein | C₁₅H₁₀O₄ | 5.60 |  | - | 253.25 | 18.0 | 223.95 | 26.0 |
|  |  | Glycitein | C₁₆H₁₂O₅ | 5.78 |  | - | 283.10 | 20.0 | 267.85 | 19.0 |
|  |  | Calycosin | C₁₆H₁₂O₅ | 5.92 |  | - | 283.40 | 20.0 | 267.85 | 20.0 |
|  |  | Genistein | C₁₅H₁₀O₅ | 6.64 |  | - | 269.25 | 19.0 | 132.90 | 29.0 |
|  |  | Formononetin | C₁₆H₁₂O₄ | 7.54 |  | - | 267.30 | 19.0 | 251.95 | 21.0 |
|  |  | Biochanin A | C₁₆H₁₂O₅ | 8.80 |  | - | 283.10 | 20.0 | 267.90 | 21.0 |
|  | Anthocyanin  (-chloride) | Cyanidin 3-O-ß-galactoside | C₂₁H₂₁O₁₁^+^(CI^-^) | 2.44 |  | + | 448.85 | -13.0 | 287.00 | -22.0 |
|  |  | Cyanidin 3-O-ß-glucoside | C₂₁H₂₁O₁₁^+^(CI^-^) | 2.44 |  | + | 448.80 | -22.0 | 287.00 | -21.0 |
|  |  | Malvidin-3-O-glucoside | C₂₃H₂₅O₁₂^+^(CI^-^) | 2.90 |  | + | 492.95 | -14.0 | 331.15 | -20.0 |
|  |  | Peonidin 3-O-ß glucoside | C₂₂H₂₃O₁₁^+^(CI^-^) | 2.93 |  | + | 462.90 | -23.0 | 301.05 | -20.0 |
|  |  | Pelargonidin 3-O-glucoside | C₂₁H₂₁O₁₀^+^(CI^-^) | 2.81 |  | + | 432.95 | -12.0 | 271.15 | -23.0 |
|  |  | Petunidin | C₁₆H₁₃O₇^+^(CI^-^) | 3.70 |  | + | 316.85 | -15.0 | 302.00 | -25.0 |
|  |  | Peonidin | C₁₆H₁₃O₆^+^(CI^-^) | 4.27 |  | + | 300.80 | -11.0 | 286.10 | -24.0 |
|  |  | Malvidin | C₁₇H₁₅O₇^+^(CI^-^) | 4.30 |  | + | 330.80 | -16.0 | 242.15 | -32.0 |
|  |  | Cyanidin | C₁₅H₁₁O₆^+^(CI^-^) | 6.78 |  | + | 286.90 | -14.0 | 137.00 | -33.0 |

t_R_ = retention time, CE = collision energy.

**Table S6**. Calibration curves of each phenolic compounds examined in this study.

| Class | Subclass | Compound | Regression | Linearity Range | LOD / LOQ^†^  (ng∙mL^-1^) | Repeatability*  (% RSD, n=2) | R^2^ | *p*-value |
| --- | --- | --- | --- | --- | --- | --- | --- | --- |
|  |  |  | Equation | (ng∙mL^-1^) |  |  |  |  |
| Phenolic acid | Hydroxybenzoic acid | Gallic acid | y = 1366.1x - 451.3 | 5 - 200 | 17.93 / 54.33 | 2.0 | 0.99890 | *p <* 0.001 |
|  |  | Salicylic acid | y = 6352.6x + 17136.0 | 5 - 400 | 28.89 / 87.54 | 1.0 | 0.99900 | *p <* 0.001 |
|  |  | *p*-Hydroxybenzoic acid | y = 3238.4x + 2198.9 | 5 - 100 | 4.22 / 12.78 | 4.5 | 0.99980 | *p <* 0.001 |
|  |  | Gentisic acid | y = 2921.0x - 5402.9 | 5 - 200 | 15.90 / 48.19 | 1.9 | 0.99900 | *p <* 0.001 |
|  |  | Protocatechuic acid | y = 3647.0x + 309.8 | 1 - 25 | 0.29 / 0.87 | 8.3 | 0.99999 | *p <* 0.001 |
|  |  | Ellagic acid | y = 442.5x - 63.4 | 10 - 150 | 14.33 / 43.43 | 4.0 | 0.99890 | *p <* 0.001 |
|  | Hydroxycinnamic acid | Ferulic acid | y = 890.5x + 797.5 | 5 - 50 | 12.69 / 38.45 | 10.5 | 0.99400 | *p <* 0.001 |
|  |  | Caffeic acid | y = 3599.4x + 20759.6 | 1 - 75 | 14.95 / 45.30 | 9.7 | 0.99520 | *p <* 0.001 |
|  |  | *trans*-Cinnamic acid | y = 1973.3x + 1443.5 | 1 - 75 | 4.54 / 13.76 | 6.9 | 0.99960 | *p <* 0.001 |
|  |  | Chlorogenic acid | y = 9670.4x - 2152.7 | 1 - 75 | 1.47 / 4.47 | 4.1 | 0.99996 | *p <* 0.001 |
|  |  | *p*-Coumaric acid | y = 5016.9x + 1322.3 | 1 - 50 | 3.10 / 9.39 | 8.1 | 0.99960 | *p <* 0.001 |
| Flavonoid | Flavonol | Quercetin | y = 4690.1x - 121.7 | 1 - 75 | 0.72 / 2.19 | 1.8 | 0.99999 | *p <* 0.001 |
|  |  | Kaempferol | y = 473.2x - 753.1 | 5 - 150 | 11.29 / 34.22 | 2.8 | 0.99910 | *p <* 0.001 |
|  |  | Rutin | y = 3573.2x - 802.2 | 1 - 75 | 10.40 / 31.51 | 7.5 | 0.99770 | *p <* 0.001 |
|  | Flavanol | (+)-Catechin | y = 1530.5x - 9220.3 | 10 - 600 | 49.36 / 149.59 | 4.2 | 0.99880 | *p <* 0.001 |
|  | Flavanone | Naringin | y = 2151.9x - 3055.1 | 5 - 50 | 5.77 / 17.47 | 6.3 | 0.99880 | *p <* 0.001 |
|  | Anthocyanin | Peonidin 3-O-ß glucoside (chloride) | y = 67704.4x + 4905.6 | 1 - 50 | 1.68 / 5.09 | 0.8 | 0.99990 | *p <* 0.001 |
|  |  | Cyanidin 3-O-ß glucoside (chloride) | y = 29584.5x - 9430.5 | 1 - 50 | 2.36 / 7.14 | 1.6 | 0.99980 | *p <* 0.001 |
|  |  | Pelargonidin 3-O-glucoside (chloride) | y = 66321.0x - 352057.7 | 10 - 800 | 37.42 / 113.40 | 0.8 | 0.99960 | *p <* 0.001 |

R^2^ = coefficient of determination, *p*-value = statistical significance of the slope.

† Based on the calibration curve used in this study, the limit of detection (LOD) and limit of quantification (LOQ) of vitamin B complex were calculated as following: LOD = 3.3ⅹ(SD of intercept/slope), LOQ = 10ⅹ(SD of intercept/slope).

* The repeatability meant the mean %RSD for duplicate instrumental analyses (n=2) of 19 phenolic standards used in each calibration curve range.

**Table S7**. Composition and content of vitamin B-complex (μg∙g^-1^, dw) in strawberries.

| Cultivars | Compounds | Semi | | |  | Full | | |
| --- | --- | --- | --- | --- | --- | --- | --- | --- |
|  |  | Aug | Sep | Oct |  | Aug | Sep | Oct |
| Goseul |  |  |  |  |  |  |  |  |
|  | B_1_ | 0.05 ± 0.01^ab^ | 0.07 ± 0.04^a^ | 0.02 ± 0.01^b^ |  | 0.03 ± 0.01^a^ | 0.04 ± 0.01^a^ | 0.01 ± 0.00^b^ |
|  | B_2_ | 0.31 ± 0.02^ns^ | 0.28 ± 0.04 | 0.29 ± 0.01 |  | 0.35 ± 0.01^ns^ | 0.28 ± 0.03 | 0.34 ± 0.05 |
|  | B_3-acid_ | 0.99 ± 0.21^a^ | 0.70 ± 0.21^ab^ | 0.58 ± 0.08^b^ |  | 1.45 ± 0.12^b^ | 1.14 ± 0.13^c^ | 1.78 ± 0.16^a^ |
|  | B_3-amide_ | 1.63 ± 0.20^a^ | 1.76 ± 0.42^a^ | 0.58 ± 0.12^b^ |  | 1.74 ± 0.14^a^ | 1.33 ± 0.05^b^ | 1.47 ± 0.09^b^ |
|  | B_5_ | 15.99 ± 3.18^ns^ | 19.71 ± 4.36 | 18.13 ± 0.98 |  | 8.19 ± 0.49^ns^ | 9.39 ± 1.65 | 7.92 ± 1.10 |
|  | B_6_ | 0.18 ± 0.01^ns^ | 0.21 ± 0.03 | 0.17 ± 0.01 |  | 0.19 ± 0.03^ns^ | 0.22 ± 0.03 | 0.20 ± 0.00 |
|  | B_7_ | 0.09 ± 0.02^ns^ | 0.10 ± 0.04 | 0.08 ± 0.01 |  | 0.05 ± 0.01^b^ | 0.08 ± 0.01^a^ | 0.02 ± 0.01^c^ |
|  | ∑ Vit B | 19.23 ± 3.2^ns^ | 22.83 ± 4.44 | 19.85 ± 1.18 |  | 12.00 ± 0.73^ns^ | 12.47 ± 1.66 | 11.74 ± 1.31 |
|  |  |  |  |  |  |  |  |  |
| Jangha |  |  |  |  |  |  |  |  |
|  | B_1_ | 0.06 ± 0.02^b^ | 0.16 ± 0.05^a^ | 0.04 ± 0.01^b^ |  | 0.06 ± 0.02^a^ | 0.07 ± 0.02^a^ | 0.03 ± 0.01^b^ |
|  | B_2_ | 0.3 ± 0.06^b^ | 0.34 ± 0.02^ab^ | 0.41 ± 0.02^a^ |  | 0.35 ± 0.00^ns^ | 0.35 ± 0.05 | 0.37 ± 0.02 |
|  | B_3-acid_ | 0.39 ± 0.09^ab^ | 0.27 ± 0.02^b^ | 0.49 ± 0.09^a^ |  | 1.78 ± 0.14^ns^ | 1.68 ± 1.41 | 2.65 ± 0.93 |
|  | B_3-amide_ | 1.64 ± 0.31^a^ | 1.14 ± 0.07^b^ | 0.50 ± 0.04^c^ |  | 2.01 ± 0.02^a^ | 1.55 ± 0.16^a^ | 1.01 ± 0.44^b^ |
|  | B_5_ | 19.53 ± 2.04^ns^ | 19.35 ± 1.92 | 19.28 ± 2.01 |  | 8.53 ± 1.56^ns^ | 9.38 ± 2.85 | 6.65 ± 1.48 |
|  | B_6_ | 0.14 ± 0.02^ns^ | 0.19 ± 0.02 | 0.19 ± 0.02 |  | 0.21 ± 0.01^ab^ | 0.23 ± 0.01^a^ | 0.2 ± 0.02^b^ |
|  | B_7_ | 0.09 ± 0.01^b^ | 0.14 ± 0.01^a^ | 0.09 ± 0.01^b^ |  | 0.06 ± 0.01^a^ | 0.08 ± 0.02^a^ | 0.01 ± 0.01^b^ |
|  | ∑ Vit B | 22.15 ± 1.77^ns^ | 21.6 ± 1.87 | 20.99 ± 2.08 |  | 12.99 ± 1.42^ns^ | 13.34 ± 1.39 | 10.92 ± 2.04 |
|  |  |  |  |  |  |  |  |  |
| Miha |  |  |  |  |  |  |  |  |
|  | B_1_ | 0.05 ± 0.02^b^ | 0.09 ± 0.02^a^ | 0.02 ± 0.01^b^ |  | 0.06 ± 0.03^a^ | 0.05 ± 0.01^a^ | 0.01 ± 0.00^b^ |
|  | B_2_ | 0.47 ± 0.07^b^ | 0.40 ± 0.04^a^ | 0.62 ± 0.06^a^ |  | 0.55 ± 0.06^ns^ | 0.48 ± 0.03 | 0.52 ± 0.02 |
|  | B_3-acid_ | 0.34 ± 0.02^a^ | 0.20 ± 0.07^b^ | 0.30 ± 0.02^a^ |  | 0.44 ± 0.10^b^ | 0.45 ± 0.12^ab^ | 0.68 ± 0.14^a^ |
|  | B_3-amide_ | 1.67 ± 0.18^a^ | 1.29 ± 0.05^b^ | 0.63 ± 0.07^c^ |  | 1.93 ± 0.35^a^ | 1.33 ± 0.08^b^ | 0.64 ± 0.16^c^ |
|  | B_5_ | 24.31 ± 3.11^ns^ | 26.15 ± 5.04 | 27.48 ± 0.21 |  | 14.46 ± 3.47^ns^ | 10.97 ± 1.75 | 11.71 ± 0.32 |
|  | B_6_ | 0.19 ± 0.02^b^ | 0.23 ± 0.02^a^ | 0.23 ± 0.02^ab^ |  | 0.26 ± 0.01^b^ | 0.29 ± 0.02^b^ | 0.34 ± 0.02^a^ |
|  | B_7_ | 0.14 ± 0.01^ns^ | 0.11 ± 0.03 | 0.10 ± 0.00 |  | 0.09 ± 0.02^a^ | 0.10 ± 0.01^a^ | 0.06 ± 0.01^b^ |
|  | ∑ Vit B | 27.15 ± 3.23^ns^ | 28.48 ± 5.07 | 29.38 ± 0.26 |  | 17.79 ± 3.18^ns^ | 13.68 ± 1.57 | 13.95 ± 0.38 |

^a-c^ Values with different superscripts are significantly different with month in maturity (*p* < 0.05). ns = non-significant, B_1_: Thiamine, B_2_: Riboflavin, B_3-acid_: Nicotinic acid, B_3-amide_: Niacinamide, B_5_: D-Pantothenic acid, B_6_: Pyridoxine, B_7_: Biotin, ∑ Vit B: sum of vitamin B contents.

**Table S8**. Composition and content of targeted phenolic compounds (μg∙g^-1^, dw) in strawberries.

| Cultivar |  | Semi | | |  | Full | | |
| --- | --- | --- | --- | --- | --- | --- | --- | --- |
|  |  | Aug | Sep | Oct |  | Aug | Sep | Oct |
| Goseul | ∑ Phenolic acid | 453.73 ± 227.14^a^ | 224.85 ± 44.07^ab^ | 176.64 ± 42.29^b^ |  | 333.44 ± 79.62^ns^ | 393.99 ± 99.36 | 332.94 ± 78.45 |
|  | ∑ Flavonoid | 285.08 ± 74.68^ns^ | 337.52 ± 70.51 | 321.22 ± 96.39 |  | 1063.5 ± 196.38^b^ | 659.42 ± 356.50^b^ | 2799.50 ± 402.08^a^ |
|  | ∑ Phenolic compounds | 738.81 ± 300.54^ns^ | 562.36 ± 27.76 | 497.87 ± 134.80 |  | 1396.94 ± 116.79^b^ | 1053.41 ± 414.13^b^ | 3132.43 ± 479.56^a^ |
|  |  |  |  |  |  |  |  |  |
| Jangha | ∑ Phenolic acid | 309.55 ± 61.30^ns^ | 382.40 ± 70.79 | 339.82 ± 21.57 |  | 540.71 ± 66.89^ns^ | 590.43 ± 98.66 | 549.58 ± 73.02 |
|  | ∑ Flavonoid | 297.71 ± 75.80^c^ | 428.70 ± 45.16^b^ | 680.22 ± 63.24^a^ |  | 1780.71 ± 125.19^a^ | 1438.85 ± 108.07^b^ | 1390.43 ± 31.33^b^ |
|  | ∑ Phenolic compounds | 607.19 ± 117.47^c^ | 811.11 ± 44.68^b^ | 1020.04 ± 41.87^a^ |  | 2321.42 ± 71.49^a^ | 2029.27 ± 43.65^b^ | 1940.01 ± 57.87^b^ |
|  |  |  |  |  |  |  |  |  |
| Miha | ∑ Phenolic acid | 301.36 ± 84.02^ab^ | 441.09 ± 105.79^a^ | 223.53 ± 75.79^b^ |  | 406.91 ± 118.60^ns^ | 404.20 ± 94.48 | 250.47 ± 47.21 |
|  | ∑ Flavonoid | 370.34 ± 151.21^b^ | 444.82 ± 202.33^b^ | 930.97 ± 104.24^a^ |  | 1770.06 ± 176.61^b^ | 2795.00 ± 416.81^a^ | 877.33 ± 183.57^c^ |
|  | ∑ Phenolic compounds | 671.69 ± 114.45^b^ | 885.91 ± 132.35^ab^ | 1154.5 ± 169.38^a^ |  | 2176.97 ± 242.69^b^ | 3199.20 ± 422.83^a^ | 1127.80 ± 195.96^c^ |

^a-c^ Values with different superscripts are significantly different with harvest month in maturity (*p <* 0.05). ns = non-significant, ∑ Phenolic acid = sum of phenolic acid, ∑ Flavonoid = sum of flavonoid, ∑ Phenolic compounds = sum of phenolic compounds

**Table S9.** Composition and content of targeted phenolic compounds (μg∙g^-1^, dw) in Goseul.

| Subclass | Compound | Semi | | |  | Full | | |
| --- | --- | --- | --- | --- | --- | --- | --- | --- |
|  |  | Aug | Sep | Oct |  | Aug | Sep | Oct |
| Hydroxybenzoic acid | GLA | 13.91 ± 8.15^a^ | 4.48 ± 5.87^ab^ | 2.83 ± 1.13^b^ |  | 5.36 ± 0.29^ns^ | 4.53 ± 4.59 | 6.41 ± 1.29 |
|  | SCA | 3.43 ± 1.21^ns^ | 5.63 ± 6.34 | 3.42 ± 1.14 |  | 2.63 ± 2.08^ns^ | 9.05 ± 8.86 | 9.21 ± 4.29 |
|  | PHA | 2.28 ± 1.75^ns^ | 1.31 ± 0.43 | 0.84 ± 0.19 |  | 4.06 ± 2.97^ns^ | 5.01 ± 2.42 | 4.46 ± 0.89 |
|  | GTA | 3.29 ± 2.04^ns^ | 4.40 ± 2.52 | 2.25 ± 0.79 |  | 2.19 ± 2.11^b^ | 9.02 ± 4.74^a^ | 4.12 ± 1.57^ab^ |
|  | PCA | 0.39 ± 0.15^ns^ | 0.56 ± 0.16 | 0.48 ± 0.13 |  | 0.55 ± 0.44^ns^ | 0.98 ± 0.49 | 1.24 ± 0.58 |
|  | EA | 362.23 ± 179.46^ns^ | 192.77 ± 27.65 | 151.34 ± 33.40 |  | 246.54 ± 61.45^ns^ | 325.75 ± 77.50 | 191.42 ± 110.61 |
| ∑ Hydroxybenzoic acid | | 385.54 ± 190.48^ns^ | 209.14 ± 43.3 | 161.16 ± 33.80 |  | 261.33 ± 68.59^ns^ | 354.34 ± 110.34 | 216.85 ± 105.28 |
| Hydroxycinnamic acid | FRA | 3.19 ± 1.55^a^ | 1.10 ± 0.80^b^ | 0.91 ± 0.25^b^ |  | 1.33 ± 0.17^ns^ | 0.79 ± 0.59 | 1.13 ± 0.19 |
|  | CFA | 3.35 ± 2.13^a^ | 0.90 ± 1.48^ab^ | 0.43 ± 0.19^b^ |  | 2.19 ± 0.86^ab^ | 1.03 ± 1.21^b^ | 2.41 ± 0.51^a^ |
|  | tCA | 7.13 ± 2.77^ns^ | 5.96 ± 27.78 | 6.00 ± 3.94 |  | 21.56 ± 5.76^b^ | 24.33 ± 27.29^b^ | 90.82 ± 28^a^ |
|  | CGA | 0.69 ± 0.11^ab^ | 0.93 ± 0.45^ab^ | 0.50 ± 0.02^b^ |  | 0.39 ± 0.19^ns^ | 0.34 ± 0.04 | 1.00 ± 0.84 |
|  | PCMA | 53.84 ± 33.19^a^ | 6.82 ± 17.73^b^ | 7.64 ± 4.96^b^ |  | 46.63 ± 6.04^a^ | 13.17 ± 11.29^b^ | 20.73 ± 3.75^b^ |
| ∑ Hydroxycinnamic acid | | 68.2 ± 39.01^a^ | 15.71 ± 5.12^b^ | 15.48 ± 9.29^b^ |  | 72.11 ± 12.01^b^ | 39.65 ± 14.46^b^ | 116.08 ± 29.54^a^ |
| Flavonol | QCT | 0.50 ± 0.20^b^ | 1.47 ± 0.45^a^ | 1.63 ± 0.16^a^ |  | 0.60 ± 0.28^b^ | 0.78 ± 0.39^b^ | 3.94 ± 1.15^a^ |
|  | KMF | 2.38 ± 0.99^b^ | 4.66 ± 0.88^a^ | 1.92 ± 0.81^b^ |  | 1.65 ± 0.60^ns^ | 3.32 ± 1.05 | 6.21 ± 3.02 |
|  | RUT | 0.26 ± 0.10^b^ | 0.99 ± 0.99^a^ | 0.71 ± 0.21^ab^ |  | 0.34 ± 0.23^b^ | 0.33 ± 0.89^b^ | 2.14 ± 0.59^a^ |
| ∑ Flavonol | | 3.13 ± 1.25^b^ | 7.13 ± 2.23^a^ | 4.26 ± 1.12^ab^ |  | 2.60 ± 1.07^b^ | 4.44 ± 5.28^ab^ | 12.29 ± 4.43^a^ |
| Flavanol | CTC | 5.73 ± 3.16^b^ | 36.95 ± 12.89^a^ | 8.13 ± 7.19^b^ |  | 4.35 ± 0.78^b^ | 34.09 ± 0.83^a^ | 14.18 ± 5.27^b^ |
| Flavanone | NRG | 1.08 ± 0.39^ns^ | 1.20 ± 0.18 | 0.84 ± 0.05 |  | 0.91 ± 0.34^ns^ | 0.68 ± 0.33 | 0.91 ± 0.21 |
| Anthocyanin | Pn3G | 0.83 ± 0.20^ns^ | 1.22 ± 0.03 | 1.37 ± 0.17 |  | 1.31 ± 0.22^b^ | 2.34 ± 0.10^a^ | 0.36 ± 0.14^b^ |
|  | Cy3G | 24.25 ± 2.72^ns^ | 27.33 ± 6.52 | 26.85 ± 4.35 |  | 76.26 ± 20.42^b^ | 53.17 ± 2.47^b^ | 128.28 ± 5.36^a^ |
|  | Pg3G | 250.05 ± 76.48^ns^ | 263.69 ± 132.45 | 279.77 ± 100.06 |  | 978.07 ± 179.22^b^ | 564.69 ± 176.39^b^ | 2643.48 ± 396.79^a^ |
| ∑ Anthocyanin | | 275.13 ± 75.54^ns^ | 292.24 ± 53.22 | 308.00 ± 102.94 |  | 1055.64 ± 195.64^b^ | 620.20 ± 353.33^b^ | 2772.12 ± 395.08^a^ |

^a-c^ Values with different superscripts are significantly different with harvest month in maturity (*p <* 0.05). ns = non-significant, GLA = Gallic acid, SCA = Salicylic acid, pHA = *p*-Hydroxybenzoic acid, GTA = Gentisic acid, PCA = Protocatechuic acid, EA = Ellagic acid, ∑ Hydroxybenzoic acid = sum of hydroxybenzoic acid, FRA = Ferulic acid, CFA = Caffeic acid, tCA = *trans*-Cinnamic acid, CGA = Chlorogenic acid, pCMA = *p*-Coumaric acid, ∑ Hydroxycinnamic acid = sum of hydroxycinnamic acid, QCT = Quercetin, KMF = Kaempferol, RUT = Rutin, ∑ Flavonol = sum of flavonol, CTC = Catechin, NRG = Naringin, Pn3G = Peonidin 3-O-ß glucoside, Cy3G = Cyanidin 3-O-ß glucoside, Pg3G = Pelargonidin 3-O-glucoside, ∑ Anthocyanin = sum of anthocyanin.

**Table S10.** Composition and content of targeted phenolic compounds (μg∙g^-1^, dw) in Jangha.

| Subclass | Compound | Semi | | |  | Full | | |
| --- | --- | --- | --- | --- | --- | --- | --- | --- |
|  |  | Aug | Sep | Oct |  | Aug | Sep | Oct |
| Hydroxybenzoic acid | GLA | 5.03 ± 0.91^ns^ | 5.26 ± 2.59 | 4.44 ± 0.90 |  | 9.57 ± 4.03^ns^ | 7.24 ± 2.98 | 6.37 ± 1.42 |
|  | SCA | 9.93 ± 5.28^b^ | 25.63 ± 4.03^a^ | 12.25 ± 3.84^ab^ |  | 9.23 ± 1.89^b^ | 16.84 ± 4.00^a^ | 9.66 ± 3.22^b^ |
|  | PHA | 0.85 ± 0.21^b^ | 1.55 ± 0.69^a^ | 1.01 ± 0.18^b^ |  | 5.39 ± 1.10^a^ | 5.99 ± 1.73^a^ | 3.01 ± 0.79^b^ |
|  | GTA | 4.16 ± 2.78^ns^ | 10.13 ± 4.15 | 4.26 ± 1.29 |  | 3.19 ± 1.04^b^ | 8.50 ± 3.40^a^ | 3.44 ± 1.30^b^ |
|  | PCA | 0.59 ± 0.64^ns^ | 0.68 ± 0.32 | 0.41 ± 0.20 |  | 0.35 ± 0.10^b^ | 0.90 ± 0.20^a^ | 0.73 ± 0.26^a^ |
|  | EA | 161.85 ± 18.59^b^ | 231.65 ± 32.05^a^ | 137.65 ± 14.66^b^ |  | 182.04 ± 29.44^ab^ | 228.56 ± 107.21^a^ | 168.93 ± 23.71^b^ |
| ∑ Hydroxybenzoic acid | | 182.41 ± 15.16^b^ | 274.9 ± 51.73^a^ | 160.02 ± 13.49^b^ |  | 209.78 ± 35.73^ab^ | 268.03 ± 35.04^a^ | 192.15 ± 30.00^b^ |
| Hydroxycinnamic acid | FRA | 2.65 ± 1.24^ns^ | 2.43 ± 0.19 | 1.87 ± 0.32 |  | 3.00 ± 0.52^a^ | 2.83 ± 0.93^ab^ | 1.98 ± 0.60^b^ |
|  | CFA | 2.22 ± 0.79^ns^ | 1.88 ± 0.09 | 1.84 ± 0.38 |  | 3.57 ± 0.19^ns^ | 3.39 ± 0.47 | 3.25 ± 0.35 |
|  | tCA | 99.00 ± 58.52^ns^ | 83.15 ± 3.36 | 147.51 ± 13.45 |  | 140.14 ± 13.06^b^ | 165.25 ± 10.01^b^ | 246.13 ± 32.75^a^ |
|  | CGA | 3.83 ± 1.37^ns^ | 3.66 ± 0.29 | 3.31 ± 0.13 |  | 1.81 ± 0.24^b^ | 2.74 ± 0.31^ab^ | 3.35 ± 0.77^a^ |
|  | PCMA | 19.43 ± 7.38^ns^ | 16.37 ± 2.59 | 25.26 ± 1.93 |  | 182.41 ± 28.57^a^ | 148.18 ± 13.95^ab^ | 102.72 ± 26.52^b^ |
| ∑ Hydroxycinnamic acid | | 127.13 ± 66.19^ns^ | 107.50 ± 32.70 | 179.8 ± 12.02 |  | 330.93 ± 38.8^ns^ | 322.39 ± 64.86 | 357.43 ± 59.15 |
| Flavonol | QCT | 0.86 ± 0.22^ns^ | 2.83 ± 0.59 | 3.31 ± 2.13 |  | 1.00 ± 0.12^b^ | 2.44 ± 0.82^a^ | 2.58 ± 0.48^a^ |
|  | KMF | 1.61 ± 0.34^b^ | 2.80 ± 1.43^a^ | 1.68 ± 0.22^b^ |  | 2.69 ± 0.55^b^ | 4.00 ± 4.36^ab^ | 5.35 ± 1.46^a^ |
|  | RUT | 0.95 ± 0.70^ns^ | 1.74 ± 0.42 | 1.66 ± 1.01 |  | 0.57 ± 0.05^b^ | 1.15 ± 0.15^a^ | 1.02 ± 0.36^ab^ |
| ∑ Flavonol | | 3.42 ± 0.76^b^ | 7.37 ± 0.54^a^ | 6.64 ± 2.96^ab^ |  | 4.26 ± 0.60^b^ | 7.59 ± 1.76^a^ | 8.96 ± 2.12^a^ |
| Flavanol | CTC | 7.99 ± 8.21^ns^ | 16.23 ± 21.35 | 4.13 ± 0.88 |  | 2.62 ± 1.01^b^ | 12.96 ± 9.62^a^ | 4.79 ± 0.53^b^ |
| Flavanone | NRG | nd | nd | nd |  | nd | nd | nd |
| Anthocyanin | Pn3G | 0.38 ± 0.17^ns^ | 0.47 ± 0.42 | 0.64 ± 0.35 |  | 0.55 ± 0.13^ns^ | 0.69 ± 0.83 | 0.82 ± 0.31 |
|  | Cy3G | 22.45 ± 18.47^ns^ | 23.65 ± 6.67 | 27.15 ± 12.92 |  | 38.63 ± 5.99^b^ | 47.00 ± 34.13^b^ | 76.10 ± 16.62^a^ |
|  | Pg3G | 263.41 ± 88.05^b^ | 380.97 ± 46.31^b^ | 641.65 ± 60.36^a^ |  | 1734.65 ± 121.6^a^ | 1370.61 ± 319.54^b^ | 1299.77 ± 33.83^b^ |
| ∑ Anthocyanin | | 286.24 ± 79.15^b^ | 405.10 ± 31.46^b^ | 669.45 ± 62.33^a^ |  | 1773.83 ± 125.29^a^ | 1418.3 ± 112.72^b^ | 1376.68 ± 33.77^b^ |

^a-c^ Values with different superscripts are significantly different with harvest month in maturity (*p <* 0.05). nd = non detected, ns = non-significant, GLA = Gallic acid, SCA = Salicylic acid, pHA = *p*-Hydroxybenzoic acid, GTA = Gentisic acid, PCA = Protocatechuic acid, EA = Ellagic acid, ∑ Hydroxybenzoic acid = sum of hydroxybenzoic acid, FRA = Ferulic acid, CFA = Caffeic acid, tCA = *trans*-Cinnamic acid, CGA = Chlorogenic acid, pCMA = *p*-Coumaric acid, ∑ Hydroxycinnamic acid = sum of hydroxycinnamic acid, QCT = Quercetin, KMF = Kaempferol, RUT = Rutin, ∑ Flavonol = sum of flavonol, CTC = Catechin, NRG = Naringin, Pn3G = Peonidin 3-O-ß glucoside, Cy3G = Cyanidin 3-O-ß glucoside, Pg3G = Pelargonidin 3-O-glucoside, ∑ Anthocyanin = sum of anthocyanin.

**Table S11**. Composition and content of targeted phenolic compounds (μg∙g^-1^, dw) in Miha.

| Subclass | Compound | Semi | | |  | Full | | |
| --- | --- | --- | --- | --- | --- | --- | --- | --- |
|  |  | Aug | Sep | Oct |  | Aug | Sep | Oct |
| Hydroxybenzoic acid | GLA | 11.02 ± 5.87^a^ | 8.24 ± 1.70^ab^ | 3.46 ± 0.67^b^ |  | 13.03 ± 4.59^a^ | 6.67 ± 1.65^b^ | 4.65 ± 0.92^b^ |
|  | SCA | 8.71 ± 6.34^b^ | 20.00 ± 2.87^a^ | 4.97 ± 0.03^b^ |  | 14.46 ± 8.86_a_ | 13.27 ± 3.02^ab^ | 2.75 ± 0.43^b^ |
|  | PHA | 1.25 ± 0.43^b^ | 2.89 ± 0.18^a^ | 1.21 ± 0.20^b^ |  | 6.49 ± 2.42^a^ | 7.85 ± 1.44^a^ | 2.99 ± 0.48^b^ |
|  | GTA | 3.72 ± 2.52^b^ | 13.16 ± 3.3^a^ | 2.13 ± 0.12^b^ |  | 7.27 ± 4.74^ns^ | 7.25 ± 2.49 | 1.72 ± 0.22 |
|  | PCA | 0.45 ± 0.16^b^ | 1.17 ± 0.12^a^ | 0.41 ± 0.10^b^ |  | 1.00 ± 0.49^ns^ | 1.07 ± 0.20 | 1.09 ± 0.35 |
|  | EA | 177.32 ± 27.65^ns^ | 307.07 ± 115.04 | 156.2 ± 67.72 |  | 185.59 ± 77.50^ns^ | 217.51 ± 66.33 | 164.96 ± 38.66 |
| ∑ Hydroxybenzoic acid | | 202.47 ± 40.97^ab^ | 352.53 ± 119.92^ab^ | 168.34 ± 68.81^b^ |  | 227.84 ± 95.79^ns^ | 253.71 ± 66.76 | 178.10 ± 40.09 |
| Hydroxycinnamic acid | FRA | 2.18 ± 0.8^ns^ | 1.86 ± 0.89 | 1.24 ± 0.19 |  | 3.31 ± 0.59^a^ | 2.90 ± 1.21^a^ | 0.70 ± 0.28^b^ |
|  | CFA | 2.50 ± 1.48^ns^ | 2.14 ± 1.01 | 1.27 ± 0.53 |  | 4.61 ± 1.21^a^ | 4.32 ± 2.15_a_ | 1.10 ± 0.34^b^ |
|  | tCA | 58.96 ± 27.78^ns^ | 63.16 ± 5.36 | 44.65 ± 4.08 |  | 112.32 ± 27.29^a^ | 89.54 ± 23.55^a^ | 28.85 ± 11.03^b^ |
|  | CGA | 1.17 ± 0.45^ns^ | 0.93 ± 0.23 | 1.50 ± 0.78 |  | 0.50 ± 0.04^ns^ | 0.73 ± 0.17 | 0.48 ± 0.34 |
|  | PCMA | 34.07 ± 17.73^ns^ | 20.28 ± 17.61 | 6.69 ± 2.02 |  | 58.33 ± 11.29^ns^ | 53.05 ± 25.04 | 41.18 ± 15.53 |
| ∑ Hydroxycinnamic acid | | 98.89 ± 47.34^ns^ | 88.56 ± 14.34 | 55.19 ± 7.41 |  | 179.07 ± 32.15^a^ | 150.49 ± 41.52^a^ | 72.36 ± 26.55^b^ |
| Flavonol | QCT | 1.39 ± 0.45^b^ | 1.94 ± 0.41^a^ | 3.03 ± 0.24^b^ |  | 1.52 ± 0.39^b^ | 4.33 ± 0.80_a_ | 2.16 ± 0.86^b^ |
|  | KMF | 2.28 ± 0.88^ns^ | 4.50 ± 1.23 | 6.27 ± 5.10 |  | 2.88 ± 1.05^b^ | 6.93 ± 2.17^a^ | 2.97 ± 1.34^b^ |
|  | RUT | 2.24 ± 0.99^ns^ | 1.35 ± 0.37 | 1.83 ± 0.22 |  | 1.59 ± 0.89^ab^ | 3.20 ± 1.08^a^ | 0.77 ± 0.33^b^ |
| ∑ Flavonol | | 5.91 ± 1.66^ns^ | 7.85 ± 1.93 | 11.19 ± 5.76 |  | 5.98 ± 1.51^b^ | 14.44 ± 3.75^a^ | 5.84 ± 2.51^b^ |
| Flavanol | CTC | 10.85 ± 12.89^ns^ | 21.99 ± 8.88 | 6.35 ± 1.46 |  | 4.68 ± 0.83^b^ | 25.57 ± 6.15^a^ | 6.29 ± 2.23^b^ |
| Flavanone | NRG | 1.67 ± 0.18^a^ | 1.05 ± 0.31^b^ | 1.19 ± 0.17^ab^ |  | 1.05 ± 0.33^ns^ | 1.08 ± 0.19 | 0.69 ± 0.05 |
| Anthocyanin | Pn3G | 0.22 ± 0.03^ns^ | 0.22 ± 0.03 | 0.29 ± 0.05 |  | 0.40 ± 0.10^a^ | 0.44 ± 0.06^a^ | 2.51 ± 0.48^b^ |
|  | Cy3G | 32.50 ± 6.52^b^ | 37.97 ± 13.59^ab^ | 51.45 ± 2.19^a^ |  | 82.73 ± 2.47^ns^ | 114.24 ± 28.68 | 80.14 ± 32.97 |
|  | Pg3G | 319.17 ± 132.45^b^ | 375.58 ± 188.64^b^ | 860.45 ± 98.31^a^ |  | 1675.22 ± 176.39^b^ | 2639.17 ± 390.53^a^ | 781.76 ± 189.66^c^ |
| ∑ Anthocyanin | | 351.90 ± 138.93^b^ | 413.77 ± 202.15^b^ | 912.18 ± 99.47^a^ |  | 1758.35 ± 175.57^b^ | 2753.86 ± 418.66^a^ | 864.40 ± 180.93^c^ |

^a-c^ Values with different superscripts are significantly different with harvest month in maturity (*p <* 0.05). ns = non-significant, GLA = Gallic acid, SCA = Salicylic acid, pHA = *p*-Hydroxybenzoic acid, GTA = Gentisic acid, PCA = Protocatechuic acid, EA = Ellagic acid, ∑ Hydroxybenzoic acid = sum of hydroxybenzoic acid, FRA = Ferulic acid, CFA = Caffeic acid, tCA = *trans*-Cinnamic acid, CGA = Chlorogenic acid, pCMA = *p*-Coumaric acid, ∑ Hydroxycinnamic acid = sum of hydroxycinnamic acid, QCT = Quercetin, KMF = Kaempferol, RUT = Rutin, ∑ Flavonol = sum of flavonol, CTC = Catechin, NRG = Naringin, Pn3G = Peonidin 3-O-ß glucoside, Cy3G = Cyanidin 3-O-ß glucoside, Pg3G = Pelargonidin 3-O-glucoside, ∑ Anthocyanin = sum of anthocyanin.


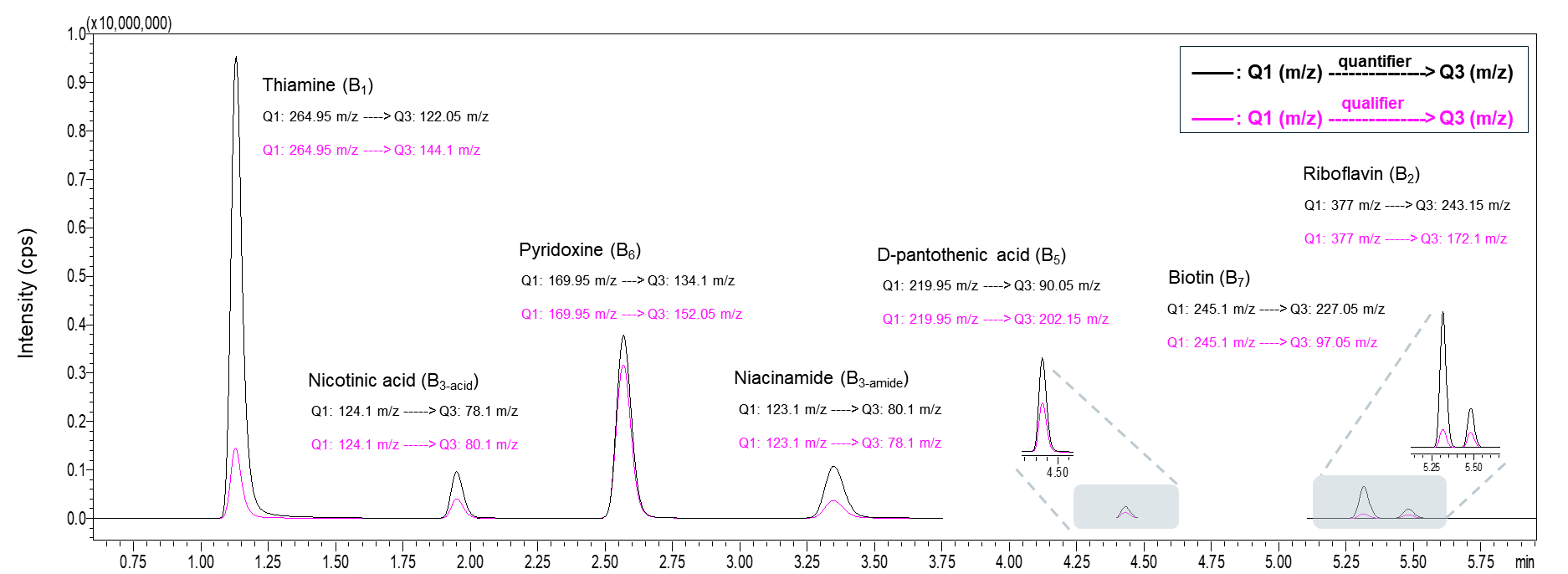


**Figure S1**. The representative ion chromatogram of vitamin B-complex standard mixture (50 ng∙mL^-1^) found in strawberry.


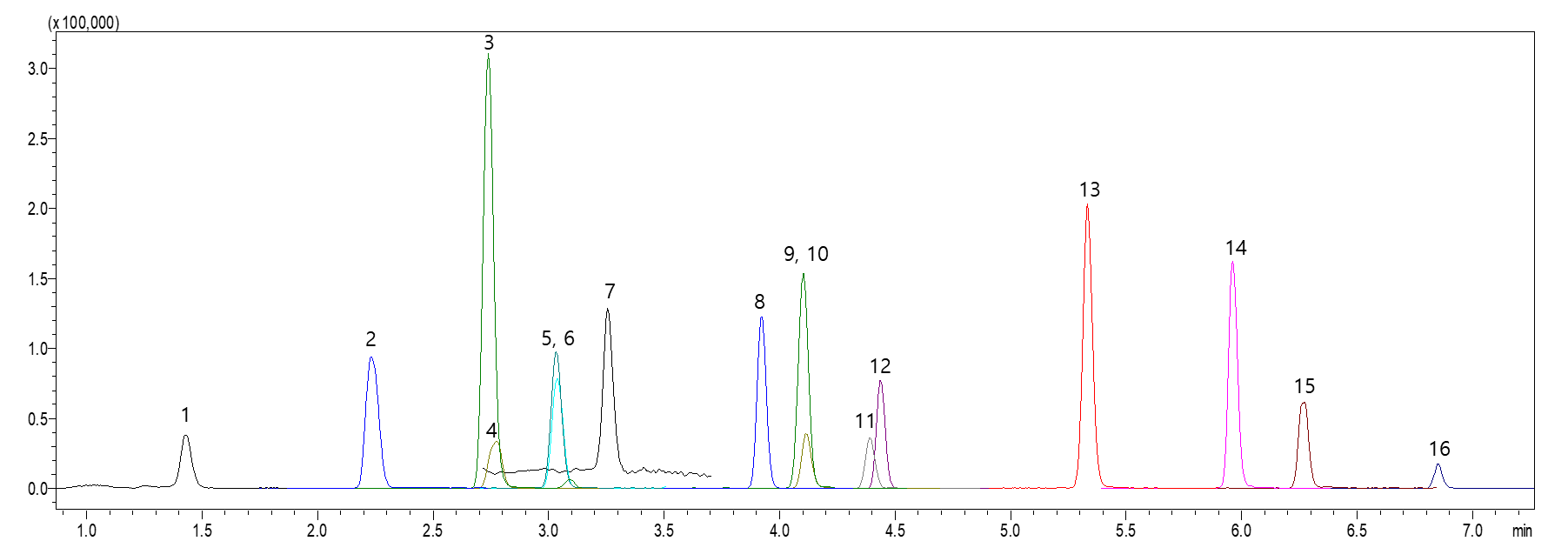
A.

Arbitrary intensity (cps)


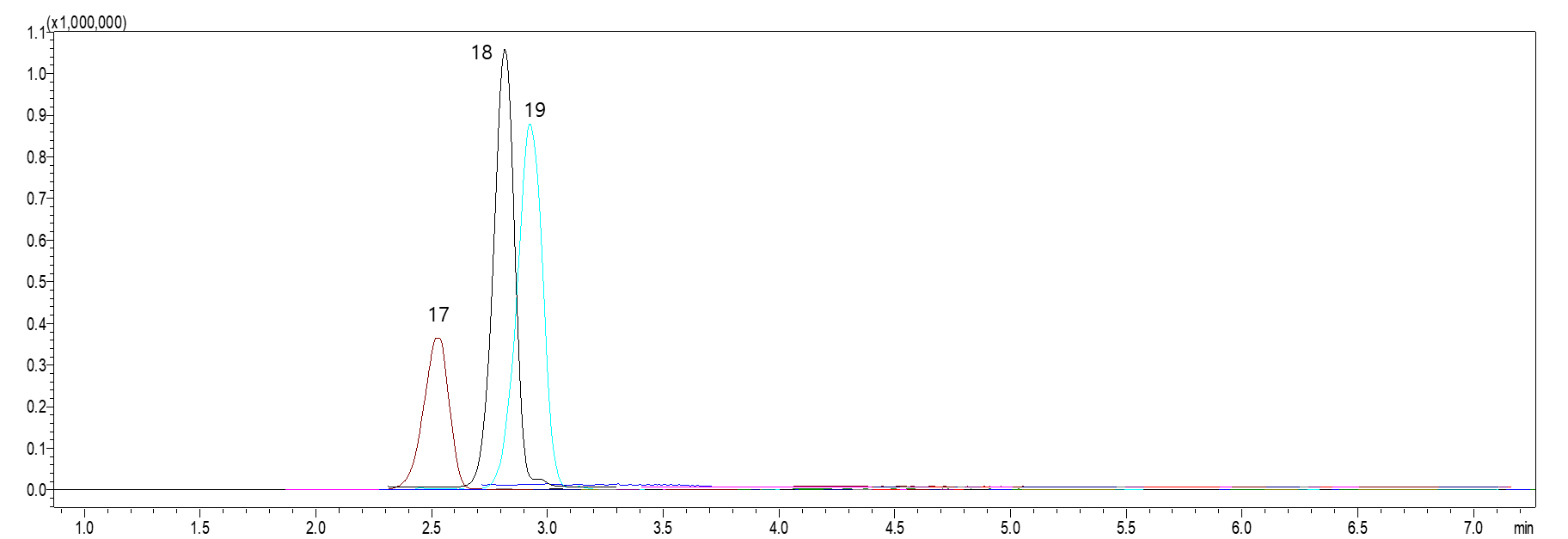


B.

Arbitrary intensity (cps)

**Figure S2**. The representative ion chromatogram of phenolic standard mixture (100 ng∙mL^-1^) found in strawberry. A: Phenolic compounds, B: Anthocyanin.

1. Gallic acid, 2. Protocatechuic acid, 3. Chlorogenic acid, 4. (+)-Catechin, 5. *p*-Hydroxybenzoic acid, 6. Gentisic acid, 7. Caffeic acid, 8. Rutin, 9. *p*-Coumaric acid, 10. Ellagic acid, 11. Ferulic acid, 12. Naringin, 13. Salicylic acid, 14. Quercetin, 15. *trans*-Cinnamic acid, 16. Kaempferol, 17. Cyanidin 3-O-ß glucoside, 18. Pelargonidin 3-O-glucoside, 19. Peonidin 3-O-ß glucoside.

**
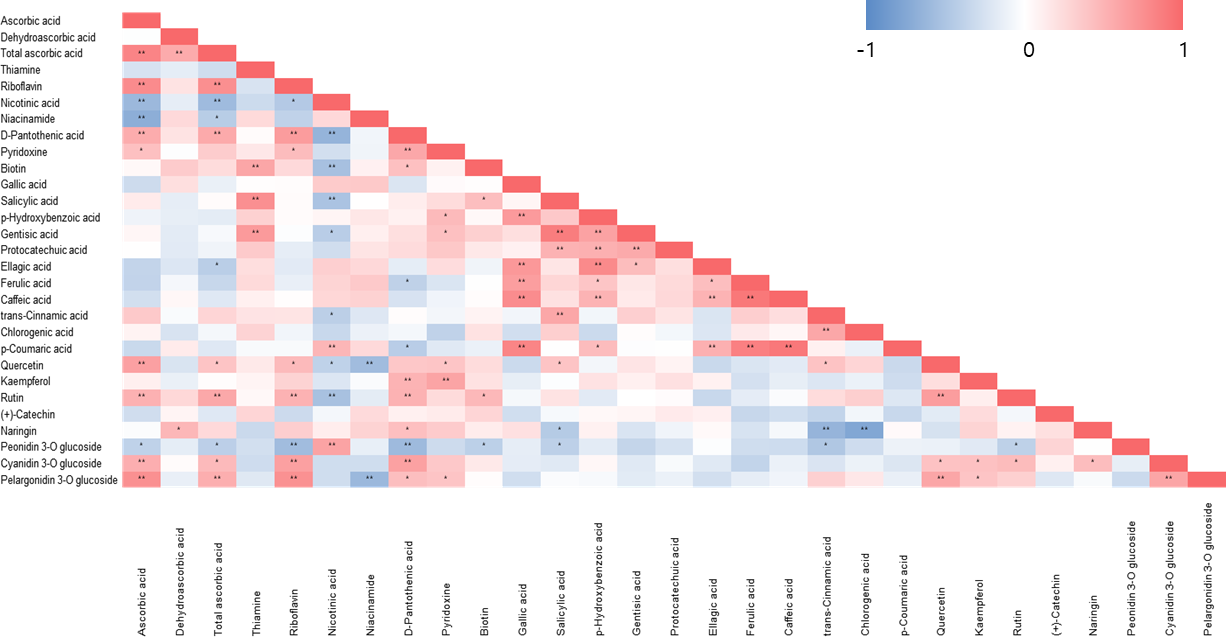
**

**Figure S3.** Correlation matrix between all detected metabolites in semi-ripened strawberries. * = *p <* 0.05, ** = *p <* 0.01.

**
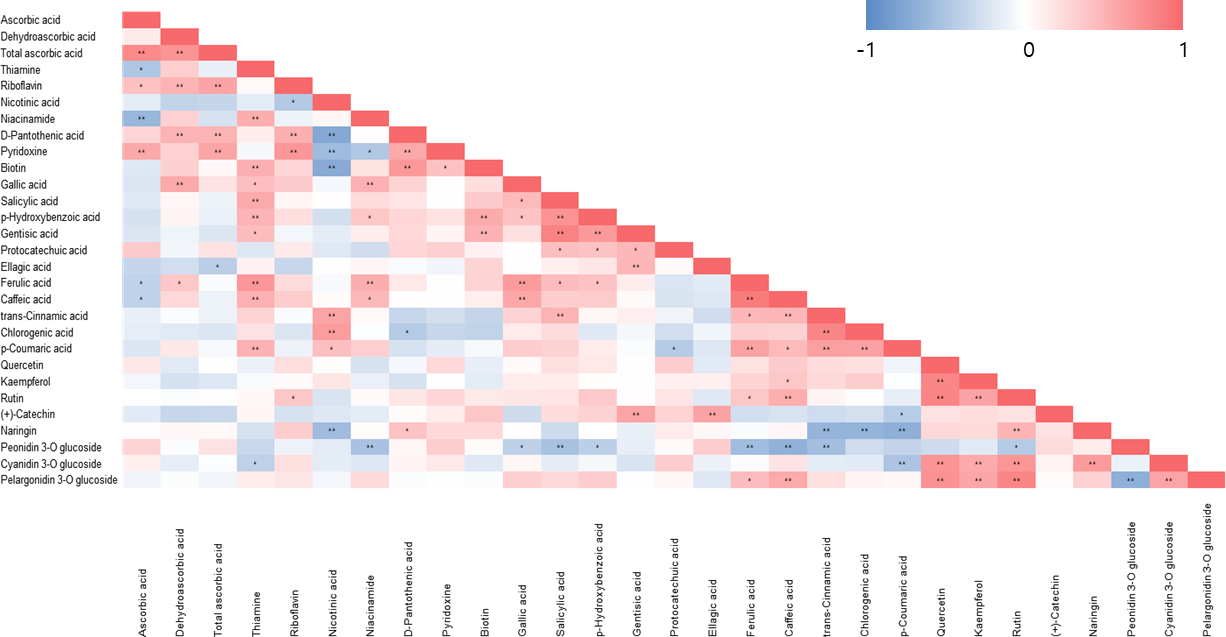
**

**Figure S4.** Correlation matrix between all detected metabolites in full-ripened strawberries. * = *p <* 0.05, ** = *p <* 0.01.
